# Genotype‐dependent contribution of CBF transcription factors to long‐term acclimation to high light and cool temperature

**DOI:** 10.1111/pce.14231

**Published:** 2021-12-06

**Authors:** Christopher R. Baker, Jared J. Stewart, Cynthia L. Amstutz, Lindsey G. Ching, Jeffrey D. Johnson, Krishna K. Niyogi, William W. Adams, Barbara Demmig‐Adams

**Affiliations:** ^1^ Department of Plant and Microbial Biology, Howard Hughes Medical Institute University of California Berkeley California USA; ^2^ Department of Ecology and Evolutionary Biology University of Colorado Boulder Colorado USA; ^3^ Molecular Biophysics and Integrated Bioimaging Division Lawrence Berkeley National Laboratory Berkeley California USA

**Keywords:** *Arabidopsis thaliana*, cold tolerance, local adaptation, photosynthetic acclimation, regulation of leaf morphology

## Abstract

When grown under cool temperature, winter annuals upregulate photosynthetic capacity as well as freezing tolerance. Here, the role of three cold‐induced C‐repeat‐binding factor (CBF1–3) transcription factors in photosynthetic upregulation and freezing tolerance was examined in two *Arabidopsis thaliana* ecotypes originating from Italy (IT) or Sweden (SW), and their corresponding CBF1–3‐deficient mutant lines it:*cbf123* and sw:*cbf123*. Photosynthetic, morphological and freezing‐tolerance phenotypes, as well as gene expression profiles, were characterized in plants grown from the seedling stage under different combinations of light level and temperature. Under high light and cool (HLC) growth temperature, a greater role of CBF1–3 in IT versus SW was evident from both phenotypic and transcriptomic data, especially with respect to photosynthetic upregulation and freezing tolerance of whole plants. Overall, features of SW were consistent with a different approach to HLC acclimation than seen in IT, and an ability of SW to reach the new homeostasis through the involvement of transcriptional controls other than CBF1–3. These results provide tools and direction for further mechanistic analysis of the transcriptional control of approaches to cold acclimation suitable for either persistence through brief cold spells or for maximisation of productivity in environments with continuous low temperatures.

## Summary Statement

Long‐term maintenance of photosynthetic upregulation was independent and freezing tolerance was partly independent of CBF1–3 in an *Arabidopsis thaliana* ecotype from a cold climate. This is contrasted with the greater reliance on CBF1–3 for long‐term acclimation in an ecotype from a warmer climate.

## 1 INTRODUCTION

Functional acclimation to cool temperatures in winter annuals has two essential components. These are activation of traits that (i) permit survival during periods of subfreezing temperatures (e.g., enhanced freezing tolerance; Kang et al., 2013; Oakley et al., 2014; Thomashow, 1999; Zhen & Ungerer, 2008) and (ii) support for continued productivity on cool days via upregulation of photosynthetic capacity, which compensates for cold‐dependent inhibition of enzymatic activity (Berry & Björkman, 1980; Cohu et al., 2014; N. P. A. Hüner et al., 1998; Savitch et al., 2002). Photosynthetic capacity is enhanced by the synthesis of greater numbers of photosynthetic proteins (Adams, Stewart, & Demmig‐Adams, 2018; Demmig‐Adams et al., 2017; N. P. Hüner et al., 1993; Stitt & Hurry, 2002; Strand et al., 1999) as well as augmentation of related features, such as the infrastructure for photosynthate export from leaves (Adams et al., 2013, 2016; Dumlao et al., 2012; Leonardos et al., 2003). Leaves of winter annuals grown in cool versus warm temperatures are also thicker and contain more chloroplast‐rich mesophyll cells per unit area (Adams et al., 2016; Cohu et al., 2014; Gorsuch et al., 2010). By virtue of upregulation of photosynthetic capacity in leaves that develop under cool temperatures (Cohu, Muller, Stewart et al., 2013), plants are able to maintain sugar production and transport for underground storage while limiting aboveground growth and exposure of leaves to freezing temperature (Eremina et al., 2016). This enhancement of photosynthesis‐related traits illustrates how acclimatory adjustment leads to new homeostasis that minimizes internal stress despite a challenging environment (Anderson et al., 1995). Notably, a similar upregulation of photosynthesis‐related features takes place during acclimation to high growth‐light intensity (Boardman, 1977; Gauhl, 1976; Munekage et al., 2015) in many species, including *Arabidopsis thaliana* (Hoshino et al., 2019; Stewart, Polutchko, Adams, Cohu, et al., 2017; Stewart, Polutchko, Adams, & Demmig‐Adams, 2017). Common regulatory networks may thus be involved in both cold and high light acclimation, such as signalling networks that respond to the level of excitation pressure in the chloroplast (Anderson et al., 1995; N. Hüner et al., 2012; N. P. A. Hüner et al., 2016).

The transcription factor family of C‐repeat‐binding factors (CBFs) has been proposed as a regulatory network that may orchestrate photosynthetic upregulation and enhance freezing tolerance in response to growth under cool temperatures and/or high light intensities (N. P. A. Hüner et al., 2016; Savitch et al., 2005). *A. thaliana* contains three tandemly duplicated *CBF* paralogs (*CBF1, CBF2* and *CBF3*; abbreviated to *CBF1–3* in this text) that are strongly induced by cold temperature and orchestrate transcriptional and physiological changes necessary for enhanced freezing tolerance (Knight & Knight, 2012; Shi et al., 2018; Thomashow, 1999). Laboratory studies revealed largely overlapping functions for the CBF1–3 transcription factors as well as a requirement for combined loss‐of‐function mutations in all three genes to strongly reduce the induction of freezing‐tolerance genes and freezing tolerance itself (Gilmour et al., 2004; Jia et al., 2016; Zhao et al., 2016). CBF overexpressing lines exhibited higher freezing tolerance as well as greater leaf thickness, chlorophyll levels and photosynthetic rates per unit area even when grown under low light and warm temperature (Gilmour et al., 2004; Savitch et al., 2005). Thus, CBF overexpression induced both the survival trait of enhanced freezing tolerance and the productivity‐maintenance trait of photosynthetic upregulation.

Following a 5‐year, reciprocal transplant investigation of two *A. thaliana* ecotypes (Ågren & Schemske, 2012), Rodasen‐47 from Sweden (SW) and Castelnuovo‐12 from Italy (IT), numerous studies provided insight into the ecophysiology and genetics underlying local adaptation in this model organism. Anatomical and physiological studies revealed that SW exhibited considerably greater foliar phenotypic plasticity in response to both growth light intensity and temperature compared to IT (Adams et al., 2014, Adams, Stewart, Polutchko, et al., 2018; Cohu, Muller, Stewart, et al., 2013; Stewart et al., 2015, 2016; Stewart, Polutchko, Adams, Cohu, et al., 2017). While possessing a similar constitutive freezing tolerance, in warm‐grown plants, SW also induced greater freezing tolerance relative to IT when grown under controlled cold conditions (Gehan et al., 2015; Park et al., 2018; Sanderson et al., 2020). Under field growth conditions, the *CBF1–3* region was identified as a QTL for fitness (Ågren et al., 2013) as well as freezing tolerance (Oakley et al., 2014). In fact, IT possesses a naturally occurring 8‐bp deletion in its *CBF2* gene that renders the CBF2 transcription factor nonfunctional (Gehan et al., 2015). Nevertheless, CBF2‐deficient lines of SW still maintained greater cold‐induced freezing tolerance than IT (Park et al., 2018; Sanderson et al., 2020). Likewise, a CBF1–3‐deficient line created in SW maintained greater cold‐induced freezing tolerance than a CBF1–3‐deficient line created in IT (Park et al., 2018).

In the present study, IT and SW were grown under a factorial design of different light intensity and temperature regimes. Transcriptome data from fully expanded leaves were generated to compare expression patterns of genes associated with the functional traits of freezing tolerance and photosynthesis and chloroplast redox state (reduction state of the primary electron acceptor of photosystem II [PSII], Q_A_) was assessed to address the relationship between chloroplast excitation pressure and *CBF1–3* expression levels. Under the two most different growth conditions, the wild‐type ecotypes, IT and SW, were grown alongside the corresponding CBF1–3‐deficient mutant lines it:*cbf123* and sw:*cbf123* (Park et al., 2018). Fully expanded leaves of these plants that had developed under the respective growth conditions were assayed for freezing tolerance, morphological and photosynthetic characteristics and expression of genes associated with the latter phenotypic traits.

## 2 MATERIALS AND METHODS

### 2.1 Plant material and growth conditions

*A. thaliana* ecotypes IT (Castelnuovo‐12 [ABRC stock number: CS98761], subline 24) and SW (Rodasen‐47 [ABRC stock number: CS98762], subline 29) were grown from seed in Conviron E15 (Controlled Environments Ltd.) and then in E36‐HID (Percival Scientific) growth chambers alongside the corresponding CBF1–3‐deficient lines it:*cbf123* and sw:*cbf123* that had been generated by Park et al. (2018) via CRISPR/Cas9 (for more information on the ecotypes, see Adams et al., 2016; Ågren & Schemske, 2012). For selected experiments, sw:*cbf2*, a CBF2‐deficient line created in SW by the same group (Park et al., 2018), was included as well. *CBF1–3* genotypes of it:*cbf123*, sw:*cbf123* and sw:*cbf2* plants used in this study were confirmed by Sanger sequencing.

The following four growth conditions—based on a factorial design of contrasting light intensities and leaf temperatures—were employed: low light, warm temperature (LLW; 9‐h photoperiod of 100 µmol photons m^−2^ s^−1^ and leaf temperature 25°C/20°C [light/dark]), low light and cool temperature (LLC; 9‐h photoperiod of 100 µmol photons m^−2^ s^−1^ and leaf temperature maximum of 16°C/12.5°C [light/dark]), high light, warm temperature (HLW; 9‐h photoperiod of 1000 µmol photons m^−2^ s^−1^ and leaf temperature 25°C/20°C [light/dark]) and high light, cool temperature (HLC; 9‐h photoperiod of 1000 µmol photons m^−2^ s^−1^ and leaf temperature maximum of 16°C/12.5°C [light/dark]), the last of which was chosen as the key condition for the present study based on previously reported phenotypic differences between IT and SW (Cohu, Muller, Demmig‐Adams et al., 2013, Cohu, Muller, Stewart, et al., 2013). The setpoints for the growth chambers were 25°C/20°C (light/dark) for LLW, 16°C/12.5°C (light/dark) for LLC, 20°C/20°C (light/dark) for HLW and 8°C/12.5°C (light/dark) for HLC. The controlled conditions chosen here are an approximation of total daily photon input in natural settings. While peak natural light intensity would be higher in exposed sites, the HL condition of 1000 µmol photons m^−2^ s^−1^ of continuous light for the duration of the light period approximates total light supply on a clear day at the point of origin for both ecotypes (Adams et al., 2016). The LL growth regime resembles peak light intensities in a shaded environment. All plants were grown from seeds soaked in water at 4°C for 4 days and then germinated in six‐pack seed‐starting trays containing 50 ml of Fafard Growing Mix 2 (Sun Gro Horticulture) under 9‐h photoperiods of either 100 (LLW and LLC) or 1000 (HLW and HLC) µmol photons m^−2^ s^−1^ and a common air temperature of 25°C during the photoperiod and 20°C during the dark period. Following germination, individual seedlings were transplanted with 50‐ml soil from their respective cells into larger (2.9 L) pots, and then transitioned to their final growth conditions (for details, see Figure S1; see also Cohu, Muller, Stewart et al., 2013). Plants received water daily with nutrients added every other day as previously described (Stewart, Polutchko, Adams, & Demmig‐Adams, 2017). Sampled plants were all nonflowering and of similar size (for plant age, see Figure S1). Unless otherwise specified, samples were taken at the end of the 15‐h dark period from young leaves that were no less than two‐thirds expanded.

### 2.2 Leaf phenotypic traits

Leaf photosynthetic capacity was determined as light‐ and CO_2_‐saturated oxygen evolution with leaf disc oxygen electrodes (Hansatech Instruments Ltd.; Delieu & Walker, 1981) as previously described (Stewart, Polutchko, Adams, & Demmig‐Adams, 2017). The reduction state of the primary electron acceptor of PSII, Q_A_, was assessed via measurements of chlorophyll fluorescence using a pulse‐amplitude‐modulated (PAM) chlorophyll fluorometer (FMS2; Hansatech Instruments Ltd.). Leaves were darkened for 20 min, exposed to a far‐red light of 0.6 µmol photons m^−2^ s^−1^ for 5 min, and then subjected to 5‐min exposures of increasing light intensities. At the end of each 5‐min exposure, steady‐state fluorescence (Strand et al., 1999) were recorded, maximum fluorescence levels (*F*_m_′) were obtained by applying a saturating pulse of light (0.8 s of 3000 µmol photons m^−2^ s^−1^) and then minimum fluorescence levels (*F*_o_′) were recorded by briefly darkening the leaf. Q_A_ reduction state was calculated as 1−q_L_ = (1/*F*_s_ − 1/*F*_m_′)/(1/*F*_o_′−1/*F*_m_′). Measurements on LLW plants were conducted in the laboratory at ambient temperature (approximately 22°C) and measurements on HLC plants were conducted inside the growth chamber in which they were grown (with an air temperature of 8°C). Chlorophyll *a* and *b* content was determined from leaf discs via high‐performance liquid chromatography as previously described (Stewart et al., 2015) or via spectrophotometry as previously described (Arnon, 1949).

Leaf dry mass was measured with an A‐160 balance (Denver Instruments Company) from leaf discs that were dried at 70°C for 7 days. For leaf‐thickness measurements, leaves were embedded in 7% (w/v) agarose and sectioned into 80–100 µm thick sections using a 752/M Vibroslice tissue cutter (Campden Instruments Ltd.). Sections were stained with 0.02% toluidine blue O for 30 s, and images were taken approximately 150 μm away from the mid‐vein (where no major veins or trichomes were present) with an AxioImager (Zeiss) coupled with a MicroPublisher color camera (QImaging). Leaf thickness was quantified for 10 representative sections of each plant (i.e., 10 technical replicates for each biological replicate) using ImageJ (Schindelin et al., 2012).

### 2.3 Freezing tolerance assays

Freezing tolerance of leaf tissue was determined via electrolyte leakage assays based on those described by Thalhammer et al. (2014). Leaves (grown under LLW or HLC conditions) with fresh‐cut petioles were placed in 300 ml of deionized H_2_O (petioles submerged) and subjected to subfreezing temperatures using an Arctic A25 refrigerated water bath (Thermo Fisher Scientific) and a cooling rate of 4°C h^−1^. Electrical conductivity was measured using an Exstik II probe (Extech Instruments). The data for each replicate were fitted to a four‐parameter logistic model and lethal freezing temperatures (LT_50_) values were determined as the inflection points from these models. Maximal intrinsic PSII efficiency in darkness was assessed in parallel with the electrolyte leakage assays after overnight incubation on ice (4°C) to thaw frozen leaves for measurements of chlorophyll fluorescence with an Imaging‐PAM Maxi (Walz). Minimal fluorescence levels (*F*_o_) were recorded after a 20‐min dark period at room temperature following the freezing treatments, and then maximal fluorescence levels (*F*_m_) were recorded by applying a pulse of saturating light (2500 µmol photons m^−2^ s^−1^). Maximal intrinsic PSII efficiency was calculated as *F*_v_/*F*_m_ = (*F*_m_ − *F*_o_)/*F*_m_, and false‐coloured images of *F*_v_/*F*_m_ were generated using ImageJ (Schneider et al., 2012).

Freezing tolerance of whole plants was determined via survival assays based on previously described protocols (Sanderson et al., 2020; Xin & Browse, 1998). Seeds were germinated and transferred to LLW or HLC growth conditions as described above with the exception that seedlings were not transferred to individual pots and were instead thinned to prevent overcrowding. After 10 days under LLW or HLC growth conditions, plants with six to eight leaves were transferred to ½ MS‐agar plates, chilled to −1°C in the presence of ice chips for 8 h, and frozen overnight (16 h) at an average freezer temperature of −10°C. To minimize positional variation in temperature in the freezer, sealed plates were put in a tray with ice before placing them into the freezer. Furthermore, the location of the three replicate plates for each genotype/growth environment pairing was randomized within the ice tray. Plates were then transferred to 4°C for 1 day, and plant survival was assessed after another 2 days of recovery in LLW conditions. Surviving plants remained green and erect, whereas nonsurviving plants were white and no longer erect.

### 2.4 Gene expression analysis of CBF1–3‐regulated genes using real‐time qPCR

RNA extraction, cDNA synthesis and qPCR were performed as previously described (Wakao et al., 2014). All primer pairs were confirmed as having 90%–105% amplification efficiency and linear amplification within their dynamic range in experimental samples using serial dilutions of cDNA before experiments. Relative transcript levels were calculated by the ΔΔ*C*_t_ method (Livak & Schmittgen, 2001) using *PEX4* (AT5G25760) as the internal reference. *PEX4*, a peroxisomal ubiquitin‐conjugating enzyme, is an established RT‐qPCR internal reference (Dekkers et al., 2011) and was confirmed in the RNAseq data set to have constant expression levels in all conditions and ecotypes. Primers were designed using Primer3 (Untergasser et al., 2012) against the 3ʹ‐UTR of each gene to avoid binding to off‐target paralogous genes. A single peak in melt‐curve analysis with a unique melting temperature was observed for each amplicon, verifying that off‐target amplification of paralogous genes was negligible.

### 2.5 RNAseq library preparation and analysis

Two flash‐frozen leaf discs of 0.73 cm^2^ were homogenized in liquid nitrogen by bead beating, and RNA was extracted and DNase‐treated using the Qiagen RNeasy Plant Mini Kit (Qiagen). Integrity of purified RNA was validated using a 2100 Bioanalyzer (Agilent Technologies) and concentration determined using a QuBit fluorometer (Thermo Fisher Scientific). Plant rRNA was depleted from 2 mg of purified RNA using the RiboZero rRNA removal kit for plants (Illumina). Barcoded cDNA libraries were generated from our rRNA‐depleted RNA samples using the TruSeq RNA library preparation kit (Illumina). Sequencing of barcoded cDNA libraries was performed at the Vincent J. Coates Genomics Sequencing Laboratory using a HiSeq 2500 platform with 50 bp single‐end reads (Illumina).

### 2.6 Statistical analyses

For phenotypic data, comparisons of two means were evaluated via Student's *t* tests and comparisons of multiple means evaluated via one‐way analysis of variance (ANOVA) coupled with post hoc Tukey–Kramer honestly significant differences (HSD) tests. The effects of genotype (e.g., CBF1–3 deficiency) and growth conditions as well as genotype response to the growth conditions for the IT (IT & it:*cbf123*) and SW (SW & sw:*cbf123*) genetic backgrounds were each assessed via two‐way ANOVA. Nonlinear curves were generated using three‐parameter exponential and four‐parameter logistic models. All statistical analyses, excluding those of RNAseq data, were conducted using JMP software (Pro 15.0.0; SAS Institute Inc.).

RNAseq statistical analysis was performed using the genomic analysis tools available through Galaxy (Afgan et al., 2018). Quality of RNAseq runs was validated by FastQC and adapter sequences were clipped using FASTQ (Gordon & Hannon, 2017). Reads were mapped to the *A. thaliana* reference genome (TAIR10) and preliminary differential expression analysis was conducted using HISAT and StringTie (Pertea et al., 2015). Differential expression analysis was conducted using DESeq. 2 as well as the calculation of adjusted *p‐*values, which limit high false positive discovery rates due to multiple testing (Love et al., 2014). Data can be accessed on the Gene Expression Omnibus at GSE154349. Log_2_ fold‐changes were transformed with the regularized log function to minimize variance caused by low expression genes, then clustered and plotted using pheatmap. In pheatmap, each sample was clustered on the horizontal axis based on the similarity of its transcriptome to the 23 other transcriptomes. On the vertical axis, individual genes were clustered based on the similarity of their expression profile across the 24 samples to the expression profile of other genes. Gene Ontology (GO) enrichment analysis was performed using PANTHER (http://pantherdb.org/) and removal of redundant GO terms was performed using REVIGO (revigo.irb.hr). Before submission to REVIGO, GO‐terms with enrichment‐values below threefold were eliminated to remove weakly enriched GO terms.

## 3 RESULTS

### 3.1 Interaction of growth environment with ecotype in shaping photosynthesis and related features, expression of *CBF1–3* genes and leaf transcriptome

#### 3.1.1 Photosynthesis and related features

**Figure 1:**
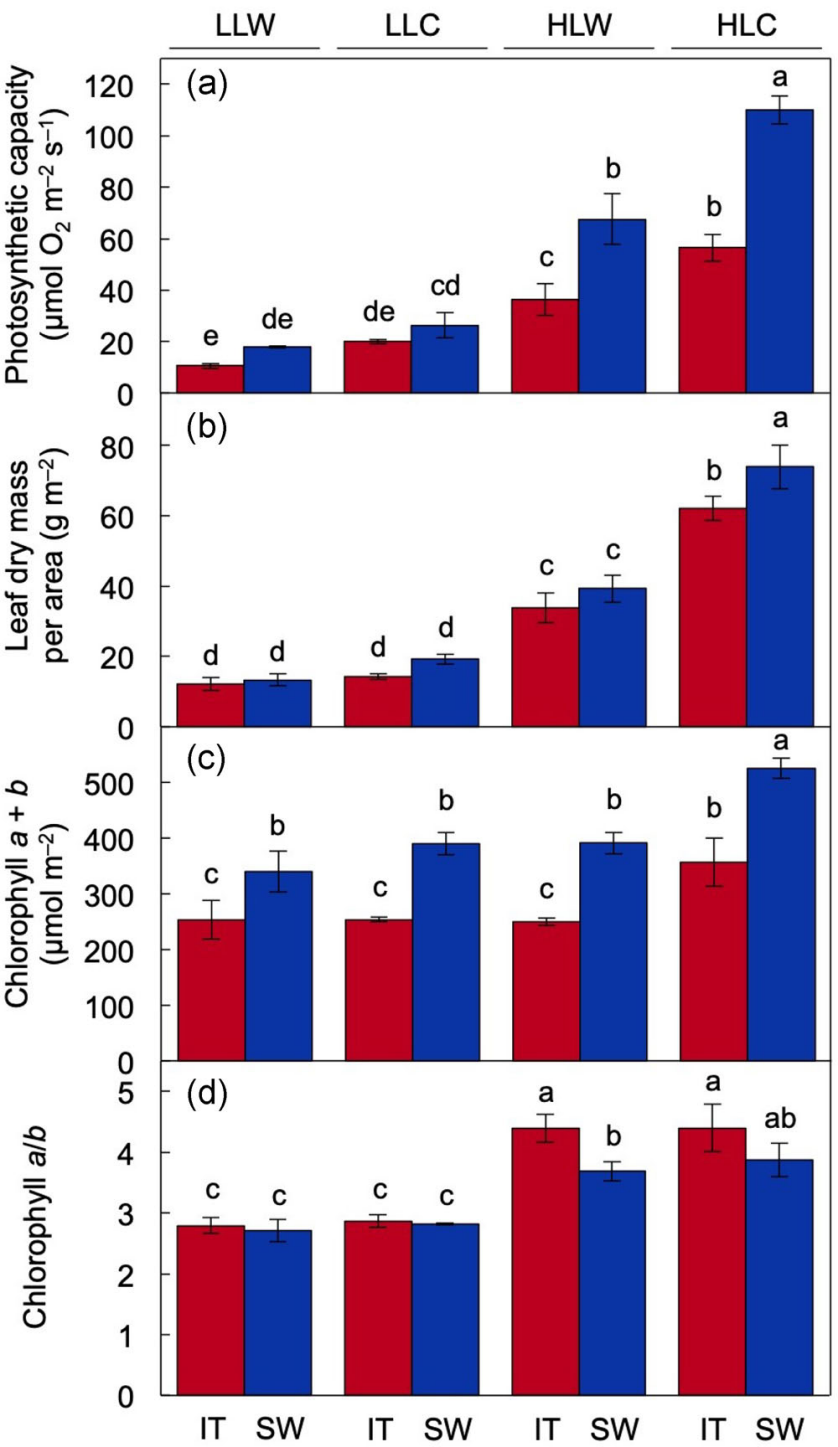
(a) Photosynthetic capacity (i.e., maximal light‐ and CO_2_‐saturated rate of oxygen evolution) per leaf area, (b) leaf dry mass per area, (c) level of chlorophyll *a* + *b* per leaf area and (d) chlorophyll *a/b* ratio in leaves of IT (red columns) and SW (blue columns) plants that were grown in low light/warm temperature growth conditions (LLW), low light/cool temperature growth conditions (LLC), high light/warm temperature growth conditions (HLW), or high light/cool temperature growth conditions (HLC). Mean values ± standard deviations (*n* = 3 or 4); groups that share the same letters are not considered statistically different, and groups that do not share the same letters are considered statistically different based on one‐way analysis of variance and post hoc Tukey–Kramer honestly significant differences tests [Color figure can be viewed at wileyonlinelibrary.com]

For both IT and SW, the highest levels of photosynthetic capacity (Figure 1a), leaf dry mass (Figure 1b) and chlorophyll *a* + *b* (Figure 1c) per leaf area were seen in plants grown under high light and cool temperature (HLC). Whereas photosynthetic capacity and leaf dry mass per area were higher in plants of both ecotypes grown under high light and warm temperature (HLW) compared to low light and warm temperature (LLW), chlorophyll *a* + *b* levels were similar. Chlorophyll *a*/*b* ratios were higher in both ecotypes in high compared to low growth light irrespective of growth temperature (Figure 1d). Significant ecotype‐specific differences were also observed, with higher photosynthetic capacity per area in SW compared to IT under HLC and HLW (Figure 1a), higher leaf dry mass per area in SW compared to IT under HLC (Figure 1b), higher chlorophyll *a* + *b* in SW compared to IT under all conditions tested (Figure 1c) and higher chlorophyll *a*/*b* ratios in IT compared to SW under HLW and a similar, albeit not significant, trend under HLC (Figure 1d).

**Figure 2:**
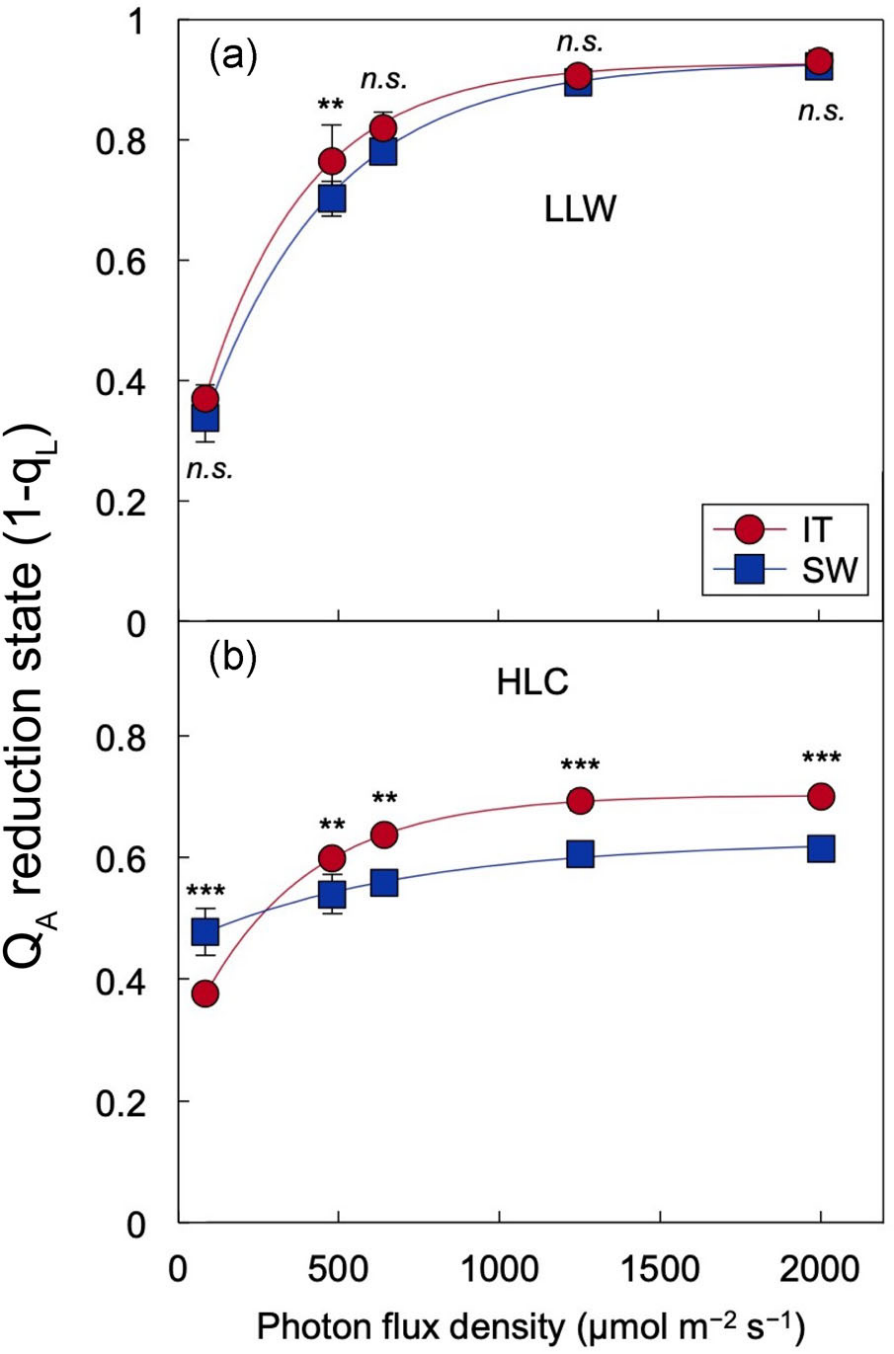
Light response of reduction state of the primary electron acceptor of photosystem II (Q_A_, quantified by chlorophyll fluorescence using the equation 1 − q_L_) of leaves from Italy (red circles) and Sweden (blue squares) plants grown under (a) LLW and (b) HLC. Mean values ± standard deviations (*n* = 3); statistically significant differences between ecotypes based on Student's *t* tests are indicated with *asterisks* (*p < 0.05, **p < 0.01, ***p < 0.001); *n.s*., not significantly different [Color figure can be viewed at wileyonlinelibrary.com]

Excitation pressure in the chloroplast was ascertained as PSII (Q_A_) reduction state after short experimental exposure to a range of light intensities in leaves of plants grown in HLC and LLW. Q_A_ reduction state was similar in the two LLW‐grown ecotypes grown across a range of light intensities (Figure 2a), with both ecotypes exhibiting a relatively steep increase to high Q_A_ reduction states with increasing light intensities. In contrast, plants of both ecotypes grown under HLC compared to LLW exhibited considerably lower Q_A_ reduction states (Figure 2a,b). Furthermore, the light response of Q_A_ reduction state differed between IT and SW plants grown under HLC, with a significantly lower Q_A_ reduction state (more oxidized Q_A_) in SW compared to IT under experimental exposure to higher light intensities (Figure 2b).

#### 3.1.2 Expression of CBF1–3 genes and leaf transcriptome

**Figure 3:**
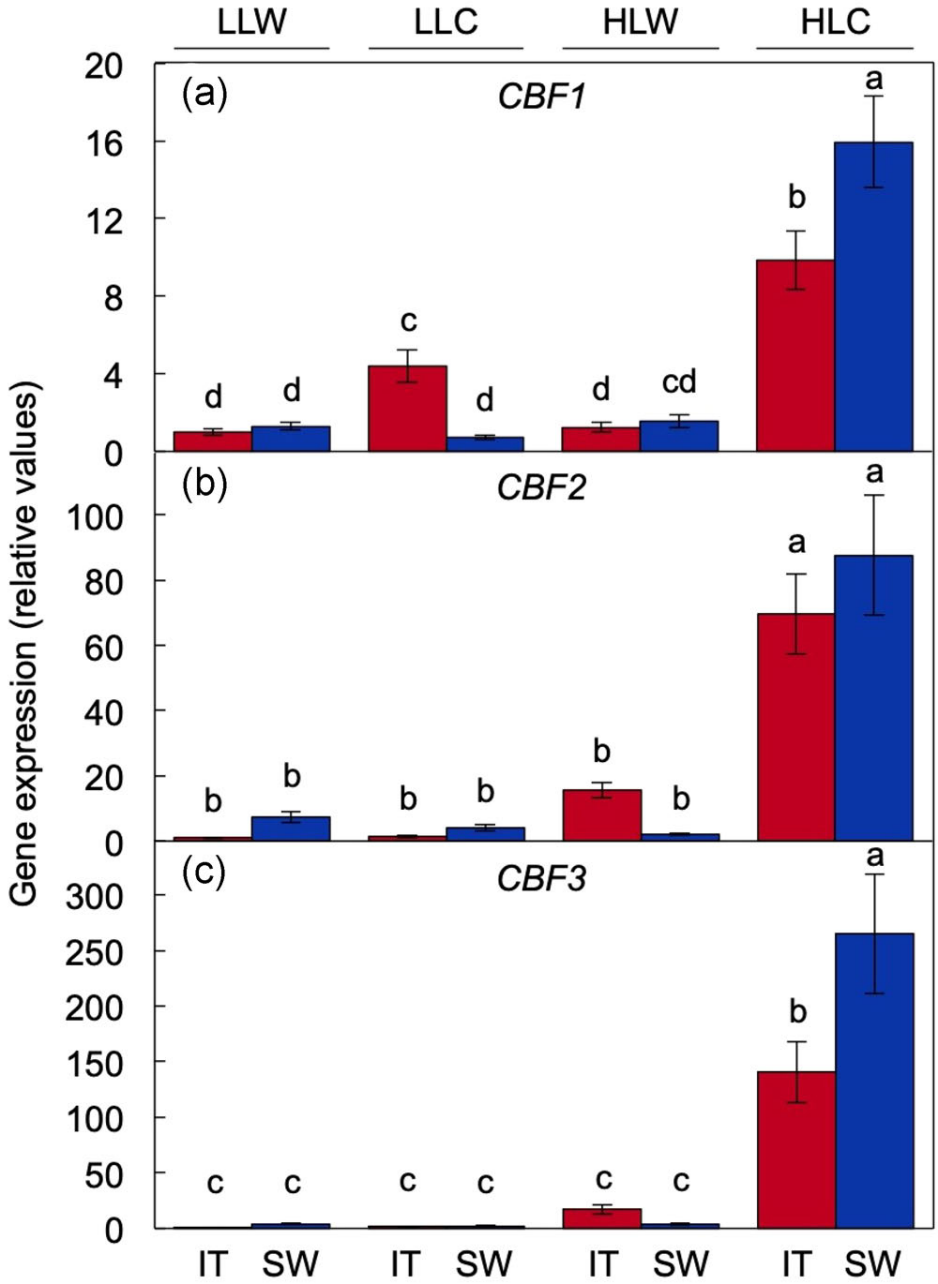
Relative transcript abundance (via RT‐qPCR) of (a) *CBF1*, (b) *CBF2* and (c) *CBF3* in leaves of IT (red columns) and SW (blue columns) plants that were grown in LLW, LLC, HLW or HLC conditions. Values are presented relative to the expression level for each respective gene in the IT ecotype grown under LLW. Mean values ± standard deviations (*n* = 3); groups that share the same letters are not considered statistically different, and groups that do not share the same letters are considered statistically different based on one‐way ANOVA and post hoc Tukey–Kramer HSD tests. IT, Italy; HLC, high light and cool temperature; HLW, high light and low temperature; LLC, low light and cool temperature; LLW, low light and warm temperature; SW, Sweden [Color figure can be viewed at wileyonlinelibrary.com]

In both ecotypes, the strongest *CBF1–3* transcript expression was also seen in plants grown under HLC (Figure 3). As was the case for photosynthetic capacity and leaf dry mass per area, *CBF1* and *CBF3* expression were also greater in SW compared to IT plants grown under HLC. In plants grown under LLC on the other hand, only IT moderately induced *CBF1–3* but SW did not (Figure 3).

**Figure 4:**
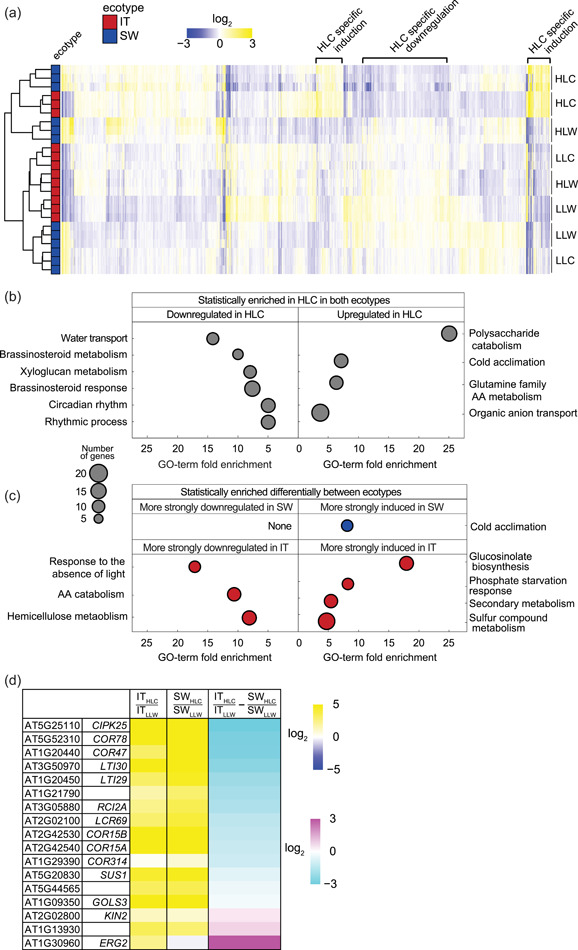
(a) Hierarchical clustering of the log_2_ expression data (via RNAseq) for 7933 genes with an adjusted *p‐*value below 0.01 in one of the pairwise comparisons for differential expression between ecotypes and growth conditions. The three biological replicates for each growth condition/ecotype set are shown as separate columns. (b) Log_2_ expression data (via RNAseq) for IT and SW in HLC relative to LLW for genes previously characterized as regulated by CBF1–3 in Col‐0 (Jia et al., 2016; Park et al., 2018). CIPK25 and KIN2 are protein kinases participating in cold acclimation signalling (Ding et al., 2019; Thomashow, 1999). COR47, LTI30 and LTI29 each are cold and drought‐induced dehydrin proteins (Puhakainen et al., 2004). SUS1 and GOLS3 are stress‐induced sucrose synthase and galactinol synthase enzymes, respectively (Maruyama et al., 2009). COR15B is essential for chloroplast membrane integrity during freezing (Thalhammer et al., 2010). (c, d) Significantly enriched gene ontology (GO) categories with a cutoff of a false discovery rate <0.05 and redundant categories removed by Revigo. GO‐term fold enrichment (i.e., how many times more frequently a gene belonging to a given GO category is identified in the set of differentially regulated genes relative to chance) is displayed on the *x*‐axis and the size of the circle is proportional to the number of genes belonging to the GO category (see the legend composed of grey circles). The significantly enriched GO categories were calculated using (c) the genes that were significantly downregulated or upregulated in both ecotypes in HLC and those (d) genes whose expression responded more strongly to HLC in one ecotype relative to the other. IT, Italy; HLC, high light and cool temperature; LLW, low light and warm temperature; SW, Sweden [Color figure can be viewed at wileyonlinelibrary.com]

Growth under HLC compared to LLW also resulted in sweeping differences in the leaf transcriptome in both ecotypes (Figure 4). A total of 1912 and 1415 genes were induced in HLC‐grown IT and SW, respectively, with an adjusted *p*‐value of less than 0.01 and a minimum fold‐change of 2 (Tables S1 and S2). Similar numbers of genes were downregulated under HLC compared to LLW, that is, 1671 and 1531 genes, for IT and SW, respectively (Tables S3 and S4). The three biological replicates cobranched (after hierarchical clustering) for both ecotypes grown under each of the four conditions, demonstrating that each of the four growth conditions elicited unique and reproducible global gene expressions in both ecotypes (Figure 4a). Genotype had a strong effect on global gene expression responses as exemplified by the cobranching of LLW and LLC datasets for ecotype (Figure 4a). The HLC growth condition had a particularly strong environmental effect on gene expression as it was the only growth condition in which IT and SW plants cobranched.

Genes induced specifically under HLC in both ecotypes were strongly enriched for several GO categories, of which the four significantly enriched unique GO terms were polysaccharide catabolism (GO:0044247), cold acclimation (GO:0009631), glutamine family amino acid metabolism (GO:0009064) and organic anion transport (GO:0015711) (Figure 4b, Tables S5–S7). Pathways repressed specifically in HLC included six with a uniqueness score below 0.1, that is, water transport (GO:0006833), brassinosteroid metabolism (GO:0016131), response to brassinosteroid (GO:0009741), circadian rhythm (GO:0007623), rhythmic process (GO:0048511) and xyloglucan metabolism (GO:0010411) (Figure 4b, Tables S8 and S9).

In addition to these shared responses, there were substantial differences between IT and SW in how strongly gene expression responded to HLC (Figure 4c, Tables S10–S16). For instance, cold acclimation genes (GO:0009631; Figure 4c,d) were consistently more strongly induced in HLC‐grown plants of SW compared to IT (Figure 4c,d, Table S11). Conversely, the four enriched and unique GO categories with significantly higher induction in HLC‐grown IT compared to SW were glucosinolate biosynthesis (GO:0019761), phosphate starvation response (GO:0016036), secondary metabolism (GO:0019748) and sulphur compound metabolism (GO:0006790) (Figure 4c, Table S14). Moreover, genes exhibiting greater downregulation in HLC‐grown plants of IT compared to SW were enriched for response to absence of light (GO:0009646), amino acid catabolism (GO:0009063), and hemicellulose metabolic process (GO:0010410) (Figure 4c, Table S16).

Genes in the photosynthesis GO category (GO:0015979) also exhibited unique expression patterns in each ecotype under HLC (Figure S2). With the exception of the light‐stress‐induced light‐harvesting complex *LHCB4.3* (AT2G40100), genes involved in light harvesting were downregulated in both ecotypes under HLC, but more so in IT relative to SW. Conversely, genes more strongly induced in IT compared to SW (Figure S2) had in common that they are typically induced under abiotic and/or oxidative stress (see Section 4), such as chloroplast glucose‐6 phosphate/phosphate translocator *GPT2* (AT1G61800), chloroplast envelope K^+^/H^+^ antiporter *KEA2* (AT4G00630), phosphofructokinase (AT1G76550), cytosolic fumarase (AT5G76550), ferritin (AT2G30400/AT3G56090) and pyridoxal phosphate synthase protein (AT5G01410).

For both ecotypes, genes preferentially induced under HLC were enriched for those that had also been induced by *CBF1–3* overexpression in the absence of either high light or cool temperatures (Park et al., 2018) (*p*‐values of 10^−19^ and 10^−18^ for IT and SW, respectively; Figure 4b, Table S17). Moreover, these genes preferentially induced under HLC were also enriched for genes expressed at lower levels in it:*cbf123* and sw:*cbf123* following sudden transfer from warm growth conditions to 4°C for 24 h (Park et al., 2018) (*p*‐values of 10^−28^ and 10^−38^, for IT and SW, respectively; Table S18). Overall, while CBF1–3 target genes (Jia et al., 2016; Park et al., 2018) were strongly induced in both ecotypes in HLC, these genes tended to be more strongly induced in SW compared to the IT ecotype in this condition. Examples for genes in this previously defined CBF1–3‐regulated group that were more strongly induced in SW included the cold acclimation‐regulating protein kinase *CIPK25* (AT5G25110) and a suite of cold‐induced dehydrin proteins, *COR47* (AT1G20440), *LTI30* (AT3G50970) and *LTI29* (AT1G20450) (Figure 4b).

Given that the LLW and HLC growth conditions had the most pronounced contrasting effect on photosynthetic capacity (Figure 1), *CBF1–3* expression (Figure 3), and the leaf transcriptome (Figure 4), we focused on comparison of these two conditions in all subsequent experiments.

### 3.2 CBF1–3 deficiency, photosynthesis, morphology, freezing tolerance and gene expression

#### 3.2.1 Photosynthesis and morphology

**Figure 5:**
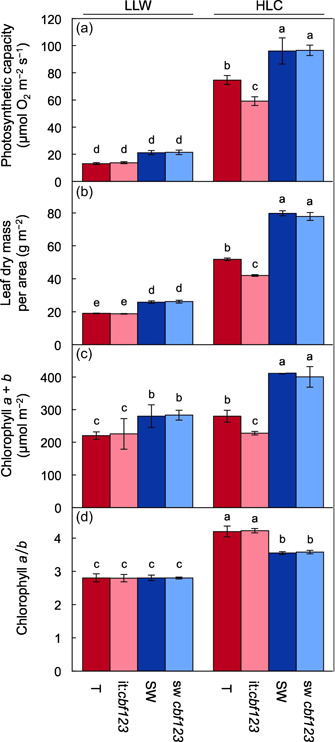
(a) Photosynthetic capacity (light‐ and CO_2_‐saturated rate of oxygen evolution) per leaf area, (b) leaf dry mass per area, (c) level of chlorophyll *a* + *b* per leaf area and (d) chlorophyll *a/b* ratio in leaves of IT (red columns), it:*cbf123* (light red columns), SW (blue columns) and (e) sw:*cbf123* (light blue columns) plants that were grown in LLW or HLC. Mean values ± standard deviations (*n* = 6); groups that share the same letters are not considered statistically different, and groups that do not share the same letters are considered statistically different based on one‐way ANOVA and post‐hoc Tukey–Kramer HSD tests. ANOVA, analysis of variance; HLC, high light and cool temperature; HSD, honestly significant differences; IT, Italy; LLW, low light and warm temperature; SW, Sweden [Color figure can be viewed at wileyonlinelibrary.com]

CBF1–3 deficiency significantly attenuated upregulation of photosynthetic capacity and leaf dry mass in HLC relative to LLW in the IT background and abolished upregulation of chlorophyll *a* + *b* content (Figure 5a–c, Table 1). Remarkably, CBF1–3 deficiency did not impede upregulation of these traits in HLC in the SW background (Figure 5a–c, Table 1). Despite the difference in chlorophyll *a* + *b* content, IT and it:*cbf123* did not differ in chlorophyll *a*/*b* under either LLW or HLC (Figure 5d).

**Table 1:** Results of two‐way ANOVAs for the effects of CBF1–3 deficiency (*cbf123*) and growth conditions as well as the interaction of these effects (*cbf123* × GC) on leaf photosynthetic capacity (Figure 5a), leaf dry mass per area (Figure 5b), and chlorophyll *a* + *b* levels (Figure 5c) and expression of associated genes (Figure 10) for the IT (i.e., IT and it:*cbf123*) and SW (i.e., SW and sw:*cbf123*) backgrounds

	IT background			SW background		
Trait or gene	*cbf123*	Growth condition	*cbf123* × GC	*cbf123*	Growth condition	*cbf123* × GC
Photosynthetic capacity	***	***	***	*n.s*.	***	*n.s*.
Leaf dry mass per area	***	***	***	*n.s*.	***	*n.s*.
Chlorophyll *a* + *b*	*	**	*	*n.s*.	***	*n.s*.
AT5G44565	***	***	***	*n.s*.	***	*n.s*.
*SUS1* (AT5G20830)	***	***	***	*n.s*.	***	*n.s*.
*LCR69* (AT2G02100)	***	***	***	*n.s*.	***	*n.s*.
AT1G13930	***	***	***	*n.s*.	***	*n.s*.
*RCI2A* (AT3G05880)	***	***	***	*	***	*n.s*.

Abbreviation: *n.s*., not significant.

*Significant effects; *p < 0.05, **p < 0.01, ***p < 0.001.

**Figure 6:**
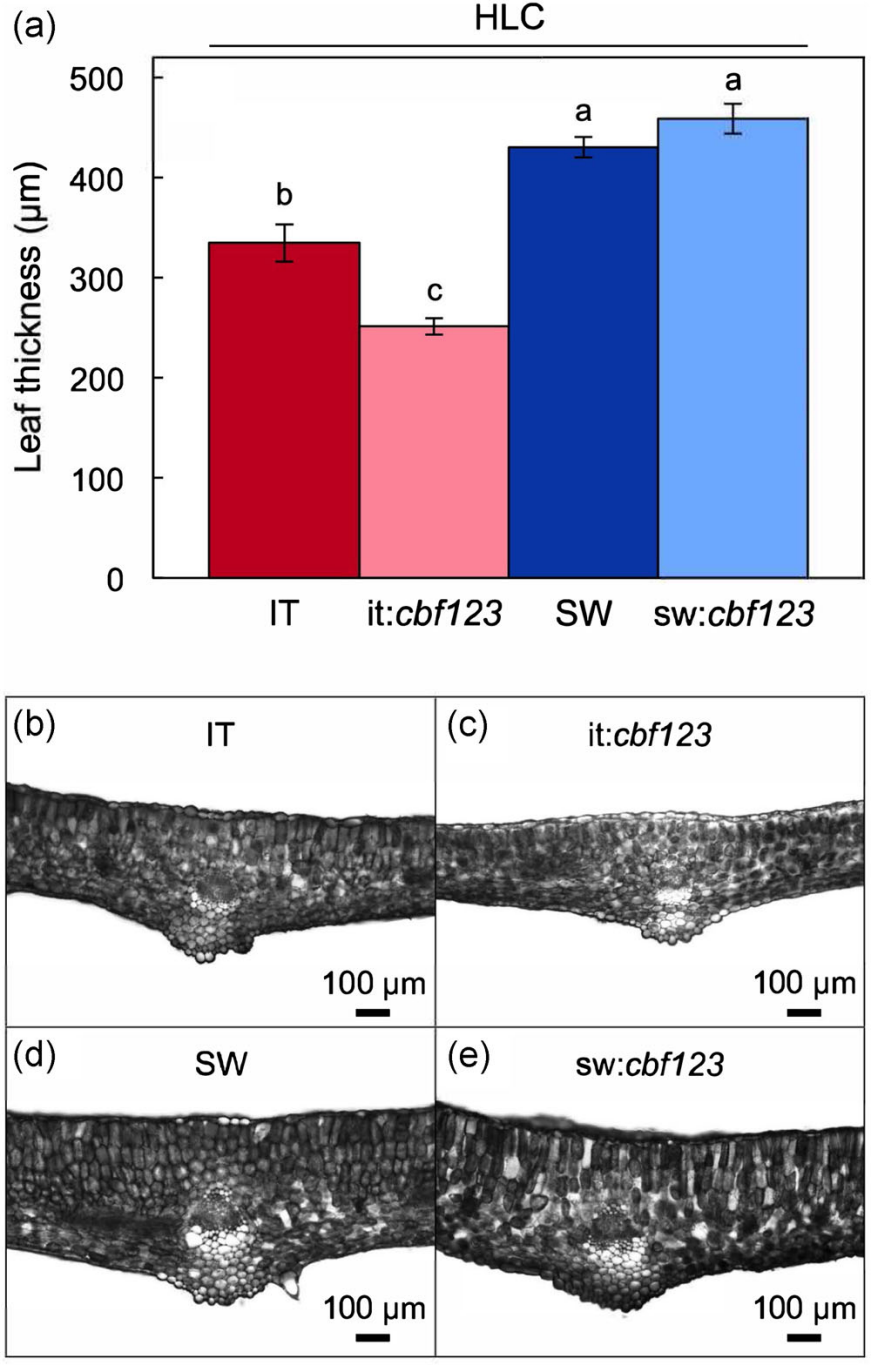
(a) Leaf thickness of IT (red column), it:*cbf123* (light red column), SW (blue column) and sw:*cbf123* (light blue column) plants that were grown in HLC, as well as representative images of leaf cross‐sections for (b) IT, (c) it:*cbf123*, (d) SW and (e) sw:*cbf123*. For (a) mean values ± standard deviations (*n* = 3); groups that share the same letters are not considered statistically different and groups that do not share the same letters are considered statistically different based on one‐way ANOVA and post‐hoc Tukey–Kramer HSD tests. ANOVA, analysis of variance; HLC, high light and cool temperature; HSD, honestly significant differences; IT, Italy; SW, Sweden [Color figure can be viewed at wileyonlinelibrary.com]

**Figure 7:**
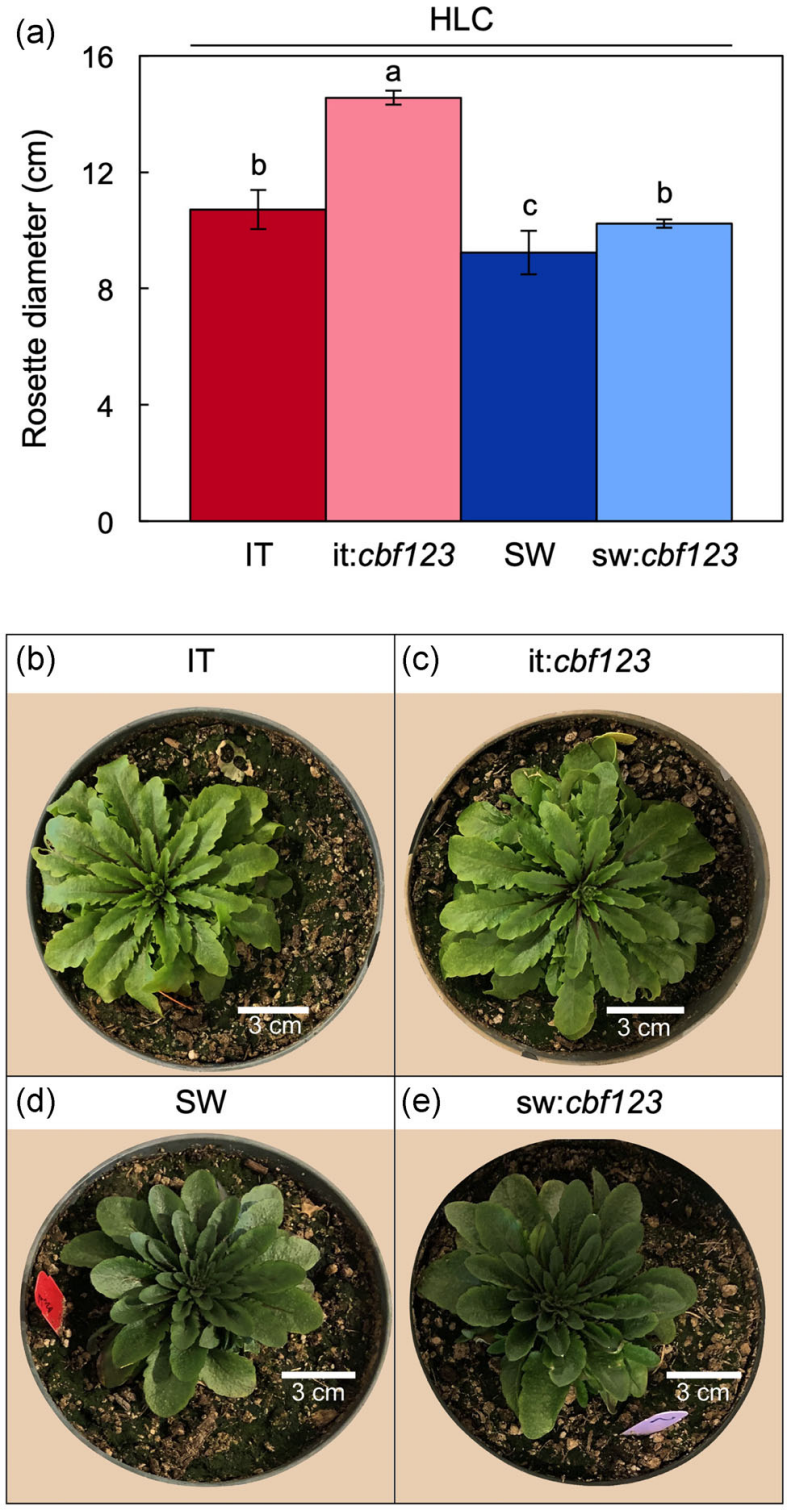
(a) Rosette diameter of IT (red column), it:*cbf123* (light red column), SW (blue column) and sw:*cbf123* (light blue column) after 40 days of growth in HLC, as well as images of representative (b) IT, (c) it:*cbf123*, (d) SW and (e) sw:*cbf123* plants. For (a) mean values ± standard deviations (*n* = 5); groups that share the same letters are not considered statistically different, and groups that do not share the same letters are considered statistically different based on one‐way ANOVA and post‐hoc Tukey–Kramer HSD tests. ANOVA, analysis of variance; HLC, high light and cool temperature; HSD, honestly significant differences; IT, Italy; SW, Sweden [Color figure can be viewed at wileyonlinelibrary.com]

Similar trends were observed for leaf morphology in that IT and it:*cbf123* grown under HLC exhibited significant differences, whereas SW and sw:*cbf123* did not (Figures 6 and 7). Specifically, leaves were thinner (Figure 6a–c) and rosettes were larger (had a larger diameter) in it:*cbf123* compared to IT (Figure 7a–c) in plants grown in HLC. In contrast, leaf thickness was the same (Figure 6a,d,e) and rosette diameter was similar in HLC‐grown plants of SW and sw:*cbf123* (Figure 7a,d,e).

#### 3.2.2 Freezing tolerance

**Figure 8:**
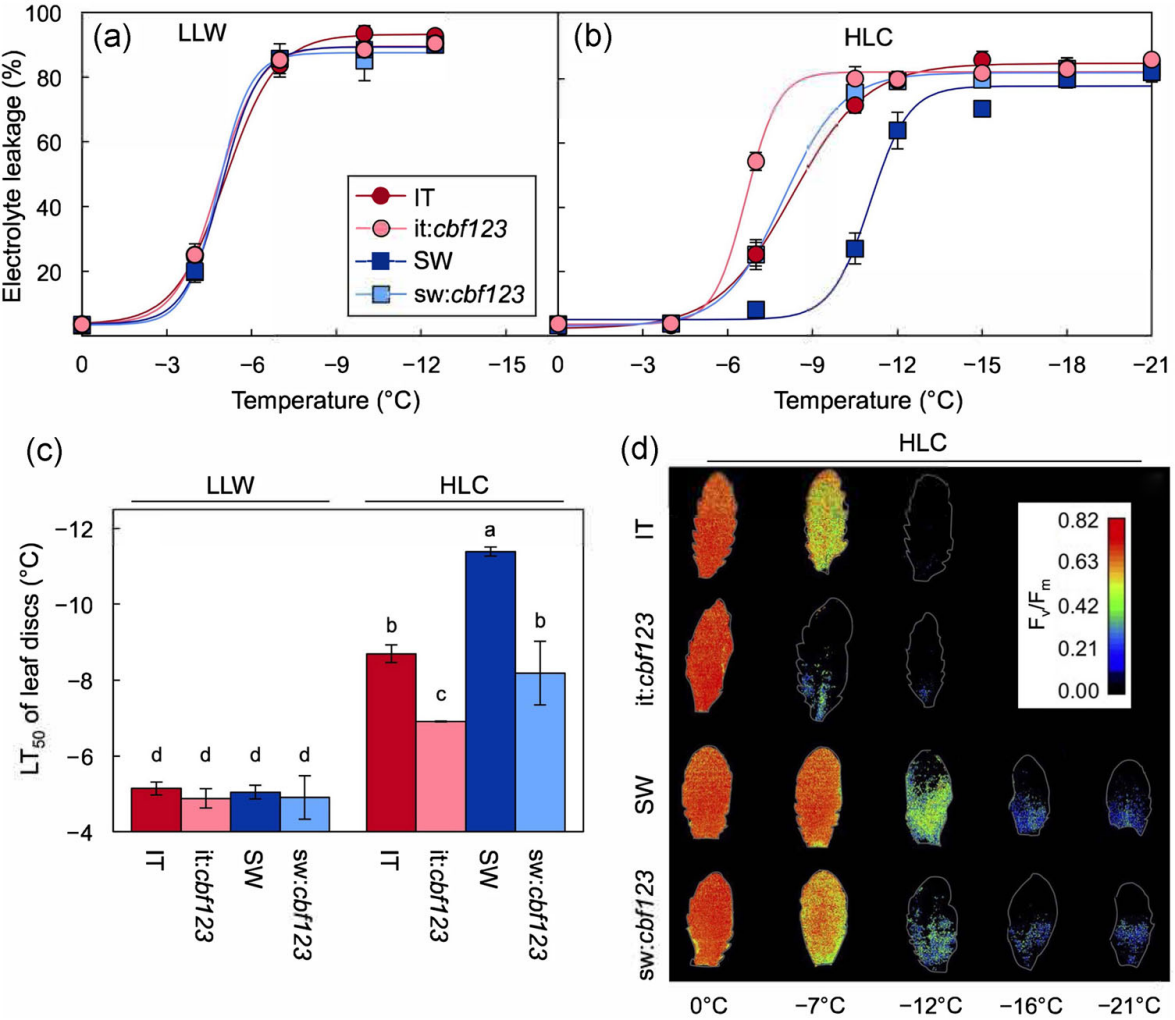
(a–c) Cellular electrolyte leakage from mature leaves, and (d) images of mature leaves with photosystem II photochemical efficiency (visualized via false colours based on *F*_v_/*F*_m_ values) from IT (red circles), it:*cbf123* (light red circles), SW (blue squares) and sw:*cbf123* (light blue squares) plants grown under LLW or HLC. For (a, b) mean values (*n* = 3). For (c), mean values ± standard deviations (*n* = 3); groups that share the same letters are not considered statistically different, and groups that do not share the same letters are considered statistically different based on one‐way ANOVA and post‐hoc Tukey–Kramer HSD tests. ANOVA, analysis of variance; HLC, high light and cool temperature; HSD, honestly significant differences; IT, Italy; LLW, low light and warm temperature; SW, Sweden [Color figure can be viewed at wileyonlinelibrary.com]

An initial assessment of leaf freezing tolerance was made using electrolyte leakage and chlorophyll fluorescence, where a sharp increase in leakage and/or decrease in intrinsic PSII indicates freezing damage to membranes (Figure 8). While LLW‐grown plants of all genotypes exhibited the same low tolerance to freezing damage by these criteria (Figure 8a), HLC‐grown plants were shifted to greater tolerance that was also more pronounced in SW compared to IT and was substantially impaired by CBF1–3 deficiency in both backgrounds (Figure 8b, Table 2). Figure 8c shows these same data transformed to mean lethal temperature (LT_50_) upon exposure to stress; onset of significant electrolyte leakage occurred with an LT_50_ near −5.6°C for all genotypes grown in LLW but was shifted to lower subfreezing temperatures in leaves grown in HLC compared to LLW to varying degrees depending on genotype. LT_50_ of freezing tolerance in sw:*cbf123* was 3.5°C warmer than that of SW (Figure 8b,c). Similarly, LT_50_ of it:*cbf123* was 3.4°C warmer than that of IT (Figure 8b,c). This greater electrolyte leakage in sw:*cbf123* compared to SW and it:*cbf123* compared to IT was accompanied by more pronounced freezing‐induced depression of intrinsic PSII efficiency *F*_v_/*F*_m_ (Figure 8d). At the same time, the lesser electrolyte leakage in both it:*cbf123* and sw:*cbf123* lines grown under HLC compared to LLW indicated contributions from CBF1–3‐independent freezing‐tolerance mechanisms.

**Table 2:** Results of two‐way ANOVAs for the effects of CBF1–3 deficiency (*cbf123*) and growth conditions as well as the interaction of these effects (*cbf123* × GC) on freezing tolerance of discs from fully expanded leaves (LT_50_; Figure 8) and immature, whole plants of six to eight leaves (% survival; Figure 9) and expression of associated genes from mature leaves (Figure 11) for the IT (i.e., IT and it:*cbf123*) and SW (i.e., SW and sw:*cbf123*) backgrounds

	IT background			SW background		
Trait or gene	*cbf123*	Growth condition	*cbf123* × GC	*cbf123*	Growth condition	*cbf123* × GC
Freezing tolerance, LT_50_	***	***	***	***	***	**
Freezing tolerance, % survival	***	***	***	*	***	*
*GolS3* (AT1G09350)	***	***	***	***	***	***
*CIPK25* (AT5G25110)	***	***	***	***	***	***
*KIN2* (AT5G15970)	**	***	**	*	***	*
*ERG2* (AT5G27930)	***	***	***	*n.s*.	***	*
*COR78* (AT5G52310)	***	***	***	***	***	***
*COR15A* (AT2G42540)	***	***	***	***	***	***
*LTI30* (AT3G50970)	***	***	***	***	***	***
*COR15B* (AT2G42530)	*	***	*	**	***	**
AT1G21790	*	***	*	*	***	*

Abbrevaition: *n.s*., not significant.

*Significant effects; *p < .05, **p < .01, ***p < .001.

**Figure 9:**
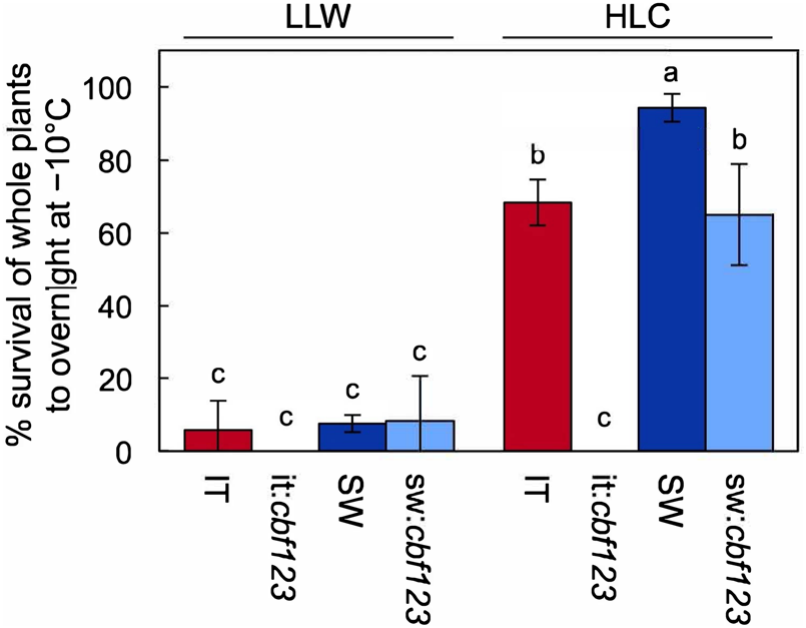
Percent survival after an overnight freezing treatment (16 h at −10°C) of IT (red columns), it:*cbf123* (light red columns), SW (blue columns) and sw:*cbf123* (light blue columns) plants grown under LLW or HLC. Mean values ± standard deviations (*n* = 3 plates, each of which contained 40 plants); groups that share the same letters are not considered statistically different, and groups that do not share the same letters are considered statistically different based on one‐way ANOVA and post‐hoc Tukey–Kramer HSD tests. ANOVA, analysis of variance; HLC, high light and cool temperature; HSD, honestly significant differences; IT, Italy; LLW, low light and warm temperature; SW, Sweden [Color figure can be viewed at wileyonlinelibrary.com]

The results from excised leaves (Figure 8) were complemented by tests of whole‐plant survival after overnight exposure to a temperature of −10°C (Figure 9). Whole‐plant survival was extremely low in LLW‐grown plants of all genotypes and was generally much enhanced by growth under HLC (Figure 9), consistent with the corresponding electrolyte leakage data obtained with leaves (Figure 8a,b). However, freezing tolerance remained severely impaired in whole plants even in HLC in it:*cbf123* plants (Figure 9). In contrast, there was much less impairment of freezing tolerance in sw:*cbf123* compared to SW for HLC‐grown whole plants (Figure 9), consistent with the trends in electrolyte leakage levels obtained for leaves (Figure 8b). A uniquely heightened sensitivity of HLC‐grown IT CBF1–3 deficient whole plants to freezing damage suggests that protective processes operating at the level of the whole plant require the presence of CBF1–3 in IT but not in SW, as is concluded here for a number of other functions.

Sample size was 120 plants per genotype/growth regime pairing for this experiment. Given that the freezing temperature of −10°C was 3.28°C below the measured LT_50_ value for IT CBF1–3‐deficient plants grown in HLC, it is possible that a sample size of more than 120 may have been necessary to see a few survivors at this freezing temperature.

#### 3.2.3 CBF1–3‐dependent gene expression

**Figure 10:**
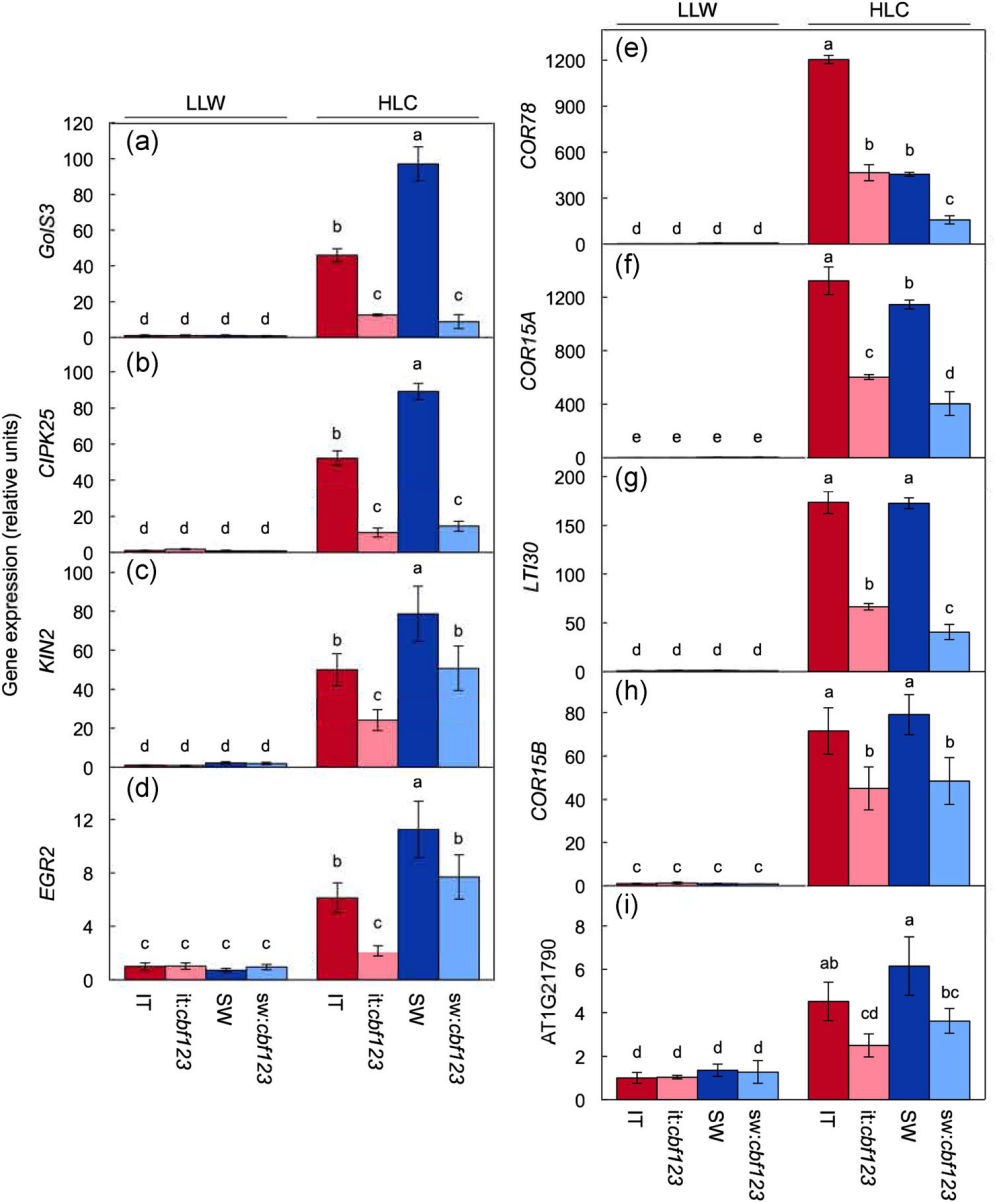
Relative transcript abundance (via RT‐qPCR) for (a) AT5G44565, (b) *SUS1*, (c) *LCR69*, (d) AT1G13930 and (e) *RCI2A* in leaves of IT (red columns), it:*cbf123* (light red columns), SW (blue columns) and sw:*cbf123* (light blue columns) plants grown in LLW or HLC. All values are normalized based on the expression levels of IT in LLW. Mean values ± standard deviations (*n* = 3); groups that share the same letters are not considered statistically different, and groups that do not share the same letters are considered statistically different based on one‐way ANOVA and post‐hoc Tukey–Kramer HSD tests. SUS1 is a stress induced sucrose synthase (Barratt et al., 2009). ANOVA, analysis of variance; HLC, high light and cool temperature; HSD, honestly significant differences; IT, Italy; LLW, low light and warm temperature; SW, Sweden [Color figure can be viewed at wileyonlinelibrary.com]

**Figure 11:**
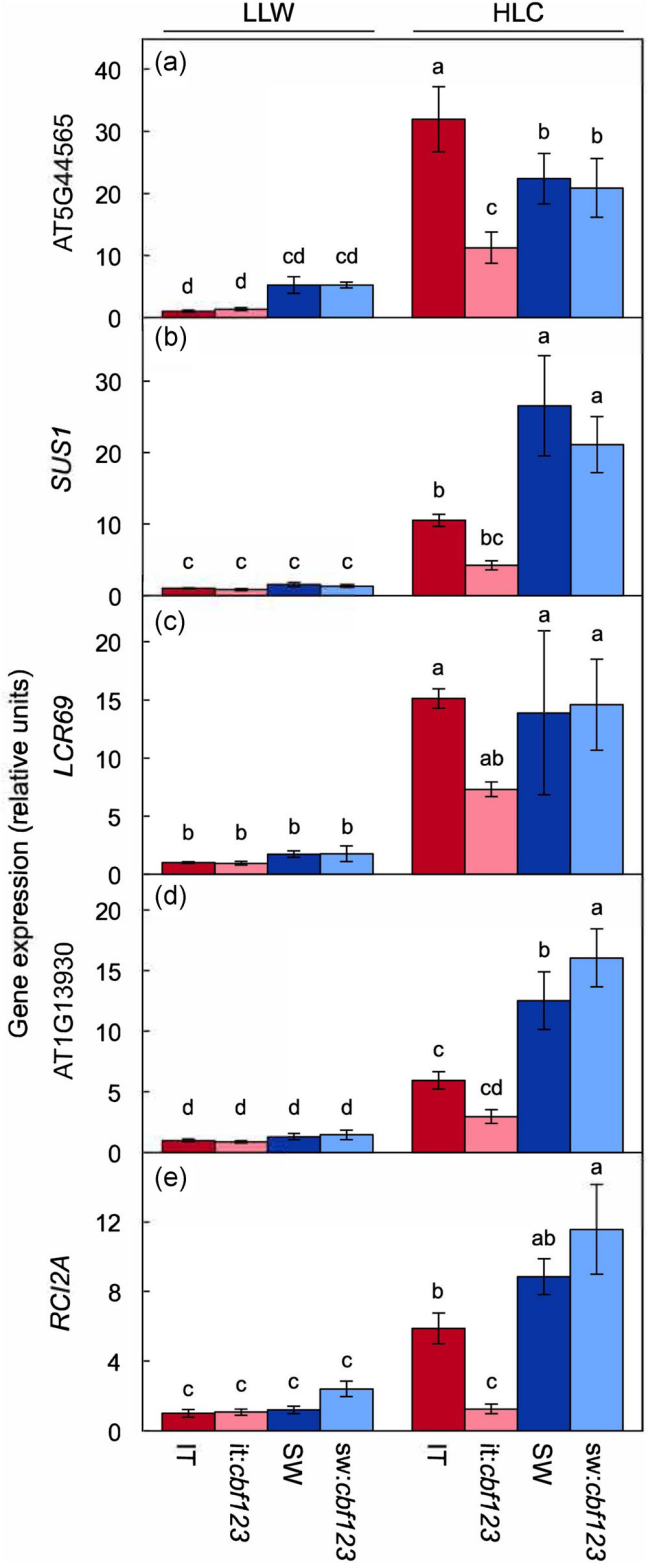
Relative transcript abundance (via RT‐qPCR) for (a) *GolS3*, (b) *CIPK25*, (c) *KIN2*, (d) *EGR2*, (e) *COR78*, (f) *COR15A*, (g) *LTI30*, (h) *COR15B* and (i) AT1G21790 in leaves of IT (red columns), it:*cbf123* (light red columns), SW (blue columns) and sw:*cbf123* (light blue columns) plants grown under LLW or HLC. CIPK25 and KIN2 are protein kinases and EGR2 is a protein phosphatase, all participating in cold acclimation signalling (Ding et al., 2019; Thomashow, 1999). LTI30 is a drought‐induced dehydrin protein (Puhakainen et al., 2004). GOLS3 is a galactinol synthase enzyme (Fowler & Thomashow, 2002). COR15B is essential for chloroplast membrane integrity during freezing (Thalhammer et al., 2010). All values are normalized based on the expression levels of IT under LLW. Mean values ± standard deviations (*n* = 3); groups that share the same letters are not considered statistically different, and groups that do not share the same letters are considered statistically different based on one‐way ANOVA and post‐hoc Tukey–Kramer HSD tests. ANOVA, analysis of variance; HLC, high light and cool temperature; HSD, honestly significant differences; IT, Italy; LLW, low light and warm temperature; SW, Sweden [Color figure can be viewed at wileyonlinelibrary.com]

This section focuses on selected genes that exhibited response patterns reminiscent of the trends exhibited by photosynthesis and leaf/plant morphology (Figure 10, Table 1) as well as selected genes known to be cold regulated (Figure 11, Table 2). From among 31 genes that were identified as CBF1–3‐target genes in prior work (Park et al., 2018) and showed considerable induction under HLC in IT (Figure 4), nine were selected for validation by RT‐qPCR with priority given to genes encoding proteins that can be linked to a role in photosynthetic or leaf‐morphological acclimation phenotypes based on either previous studies on these proteins or the presence of a protein domain with an established role in acclimation phenotypes. Expression level of five of these nine genes (Figure 10, Table 1) exhibited an impact of CBF1–3 deficiency mirroring that on leaf photosynthetic and morphological traits in the two ecotypes. Specifically, these five genes exhibited a strong reduction in the extent of upregulation under HLC compared to LLW in it:*cbf123* compared to IT but no to little difference in sw:*cbf123* compared to SW. These genes included cold‐ and salt‐responsive protein transmembrane protein AT5G44565 (Figure 10a), sucrose synthase *SUS1* (AT5G20830; Figure 10b), cysteine‐rich, defensin‐like protein *LCR69* (AT2G02100; Figure 10c) and oleosin‐B3‐like stress protein AT1G13930 (Figure 10d), *RCI2A* (AT3G05880; Figure 10e).

Moreover, expression of nine selected cold acclimation genes was affected by CBF1–3 activity in both IT and SW grown in HLC (Figure 11). Under HLC, expression of galactinol synthase *GolS3* (AT1G09350), the protein kinases *CIPK25* (AT5G25110) and *KIN2* (AT5G15970) and the protein phosphatase *EGR2* (AT5G27930) were higher in SW compared to IT and were also higher in both wildtype genotypes compared to their corresponding CBF1–3‐deficient mutants (Figure 11a–d). In contrast, expression of cold‐regulated genes *COR78* (AT5G52310) and *COR15A* (AT2G42540) was higher in IT compared to SW (Figure 11e,f), and expression of the dehydrin *LTI30* (AT3G50970), the cold‐regulated gene (necessary for chloroplast membrane integrity in freezing) *COR15B* (AT2G42530), and lipid‐sensing‐domain‐containing AT1G21790 was similar in SW and IT (Figure 11g–i). Induction of the latter genes (expressed either more strongly, or similarly, in IT compared to SW) under HLC versus LLW was associated to some extent with CBF1–3 since it was partially inhibited in sw:*cbf123* compared to SW (Figure 11, Table 2) and partially (Figure 11a–c,e–h) or completely (Figure 11d,i) inhibited in it:*cbf123* compared to IT (Table 2).

While the focus of this study was the effect of complete CBF1–3 deficiency, CBF2 deficiency alone caused a subset of CBF1–3‐regulated genes to be attenuated in HLC‐grown plants in sw:*cbf2* relative to SW (Table S19). This was associated with a small decrease in freezing tolerance of whole plants, with survivorship still higher than that of IT plants (Table S19).

## 4 DISCUSSION

### 4.1 Response of plant function and gene expression to growth under HLC conditions

Neither cool temperature alone (maximum daytime leaf temperature of 16°C) nor high light alone strongly induced expression of CBF1–3. Only combined HLC temperature had a strong effect on CBF1–3 expression. Increased excitation pressure in the chloroplast serves as one of the signals that induce elevated *CBF1–3* expression under HLC conditions (N. Hüner et al., 2012; N. P. A. Hüner et al., 2016) and is integrated with additional photosynthetic retrograde signals (Lee & Thomashow, 2012; Noren et al., 2016). Overwintering herbaceous plants, experiencing cold temperatures and the associated high excitation pressure in the chloroplast, enact the suite of acclimatory responses demonstrated here, including upregulation of photosynthetic capacity and leaf thickness (with more mass and typically, greater chlorophyll per area), reduced leaf expansion and enhanced freezing tolerance (see also Cohu et al., 2014; Cohu, Muller, Stewart et al., 2013; Muller et al., 2014; Sanderson et al., 2020). The combination of mechanisms of photosynthetic upregulation can vary given that this upregulation occurs at multiple scales (chloroplast, cell, whole leaf) that contribute differentially depending on plant genotype and environment (for a review, see Demmig‐Adams et al., 2017). These changes can allow overwintering species to achieve full acclimation, defined as new homeostasis where internal stress (with signs of oxidative stress) is minimized or absent.

The pronounced acclimation of plant form and function in SW and IT plants grown in HLC conditions was associated with sweeping changes in gene expression, with approximately 5.2% of total leaf transcriptome upregulated and 4.9% downregulated in HLC relative to growth in low light and warm temperature (LLW). The most strongly enriched and unique GO categories in induced in both ecotypes in HLC included polysaccharide catabolism, cold acclimation, glutamine family amino acid metabolism and organic anion transport, which is consistent with the upregulation of photosynthetic capacity and of freezing tolerance. Continued photosynthetic productivity under cool temperatures in the absence of significant growth generates carbohydrate that can be stored in sink tissues (Adams, Stewart, & Demmig‐Adams, 2018; Demmig‐Adams et al., 2017) and also contribute to the accumulation of compatible solutes and freezing point depression (Cao et al., 2007; Reyes‐Díaz et al., 2006; Wanner & Junttila, 1999).

Pathways repressed in HLC in both SW and IT included those associated with growth hormones. Reduction of rosette expansion under winter conditions, involving decreased rates of cell elongation during leaf development (Hoshino et al., 2019; Yano & Terashima, 2004) helps to minimize foliar freezing damage. Pathways repressed in HLC in both SW and IT included not only those associated with growth hormones (e.g., brassinosteroids) but also with water transport. In fact, vascular tissue is one of the targets of growth hormones (Etchells et al., 2017; Fàbregas et al., 2015) and acclimation to cool temperature is associated with adjustments of vascular anatomy (Adams et al., 2016; Cohu, Muller, Demmig‐Adams, et al., 2013; Stewart et al., 2016). Thus, effects of freezing on the plant vasculature may be involved in the pronounced vulnerability of it:*cbf123* plants.

Further research should address how the GO categories that are differentially expressed in the two ecotypes when grown under HLC and LLW conditions may impact the metabolome, for example, the levels of starch as well as soluble carbohydrates and other osmolytes, and regulators such as plant hormones. Furthermore, the diversity of additional GO categories that were differentially expressed in IT and SW is consistent with the range of other functions differed in these two ecotypes from different latitudes (Adams et al., 2016; Ågren & Schemske, 2012 [as a sample paper on vascular functions]). Most obviously, cold acclimation genes were consistently more strongly induced in SW in HLC conditions. Beyond this, another example was the induction of glucosinolate biosynthesis, secondary metabolism and sulphur compound metabolism genes specifically in the IT ecotype in HLC, which could be related different demands for pathogen defense at different latitudes (Roberts & Paul, 2006).

### 4.2 Differences between SW and IT in the extent of response to HLC

#### 4.2.1 Stronger enrichment in SW versus IT under HLC

Growth under HLC conditions induced cold‐acclimation genes in both ecotypes, but more strongly so in SW relative to IT. This pattern is consistent with the greater freezing tolerance and upregulation of photosynthetic capacity in SW compared to IT (see also Cohu, Muller, Stewart, et al., 2013; Stewart et al., 2016) as well as the lesser excitation pressure in the chloroplast (more oxidized Q_A_ reduction state) of HLC‐grown SW compared to IT under experimental high‐light exposure. The stronger downregulation of genes involved in light‐harvesting in HLC‐grown IT suggests that IT limits excitation pressure by lowering light‐collection capacity, which is consistent with the lower Q_A_ reduction state under very low light (when thermal dissipation is not triggered) in HLC‐grown IT compared to SW as well as IT's lower chlorophyll *a* + *b* content and higher chlorophyll *a/b* ratio that are indicative of a smaller antenna size (due to preferential degradation of the outer, chlorophyll *b*‐containing light‐harvesting complexes). This is consistent with previous studies in which SW increased, rather than decreased, light absorption during cold acclimation and apparently limited excitation pressure by greater utilisation of excitation energy in photosynthetic electron transport (Cohu, Muller, Stewart, et al., 2013), as well as greater photoprotective thermal dissipation (Oakley et al., 2018). Our present findings in HLC growth conditions indicate that the acclimatory adjustments in SW are more conducive to productivity maintenance, while adjustments in IT still mitigate oxidative stress.

Two examples of genes with expression patterns that match those of the greater photosynthetic acclimation in SW compared to IT are *SUS1* (AT5G20830) and *EGR2* (AT5G27930). *SUS1* is a sucrose synthase strongly induced under abiotic stress but not required for sucrose accumulation under conditions favourable for growth (Barratt et al., 2009; Kilian et al., 2007). High foliar sucrose levels are, furthermore, linked to increased palisade cell height in leaves grown under high light (Hoshino et al., 2019; Katagiri et al., 2016). *EGR2* is a negative regulator of growth (Bhaskara et al., 2017). Overexpression of *EGR2* caused a reduction of cell elongation and rosette size, whereas *egr2* null mutation enhanced both processes (Bhaskara et al., 2017).

#### 4.2.2 Stronger enrichment in IT compared to SW under HLC

The well‐characterized phenotypic features of cold acclimation do occur in IT, but to a lesser extent than in SW. It is noteworthy that the genes more strongly induced under HLC in IT compared to SW have been implicated in abiotic stress responses, as was reported for chloroplast glucose‐6 phosphate/phosphate translocator *GPT2* (Dyson et al., 2015), chloroplast envelope K^+^/H^+^ antiporter *KEA2* (Kunz et al., 2014), light‐harvesting complex *LHCB4.3* (Klimmek et al., 2006), cytosolic phosphofructokinase (Kant et al., 2008), cytosolic fumarase (Pracharoenwattana et al., 2010), ferritins (Petit et al., 2001) and pyridoxal phosphate synthase (Denslow et al., 2007). Future research should further test the hypothesis that both SW and IT make acclimatory adjustments that limit oxidative stress under HLC conditions, but that changes in SW focus more on the enhancement of productivity (which also lowers excitation pressure more effectively), while IT undergoes alternative evasive changes that are somewhat less effective in controlling excitation pressure.

Moreover, genes exhibiting greater downregulation in HLC in IT compared to SW were those involved in growth and signalling (response to the absence of light and hemicellulose metabolism). As stated above, such effects could be related to the hormonal control of vascular acclimation.

### 4.3 CBF1–3 involvement in acclimation to HLC conditions

#### 4.3.1 Extent of CBF1–3 involvement in SW relative to IT

The present finding that CBF1–3 are necessary for full induction of freezing tolerance in SW and IT demonstrates their involvement in *A. thaliana* grown from seedling stage in HLC conditions as done here. Previous studies had shown that CBF1–3 are required for full induction of freezing tolerance in mature plants grown under warm conditions and transferred in one step to chilling conditions (Jia et al., 2016; Park et al., 2018; Zhao et al., 2016). However, as was also concluded from studies on warm‐grown CBF1–3‐deficient mutants abruptly transferred to cold conditions (Jia et al., 2016; Park et al., 2018; Zhao et al., 2016), both CBF1–3‐dependent and CBF1–3‐independent pathways contribute to freezing tolerance in plants grown from seedling stage under HLC. The parallel result is illustrated here by the fact that freezing tolerance of both it:*cbf123* and sw:*cbf123* was greater in HLC compared to LLW and that the induction of genes previously defined as CBF1–3‐target genes was reduced to varying degrees, but was not fully blocked in CBF1–3‐deficient lines grown under HLC. The response to sudden exposure to freezing temperature in HLC‐grown wild type and CBF1–3‐deficient lines closely resembled that of plants of the same genotypes after a two‐week‐long exposure to colder, near‐freezing temperatures (air temperature = 4°C in low light, Park et al., 2018). This is noteworthy since the HLC conditions used here involve only cool temperatures (maximum leaf temperature = 16°C, air temperature = 8.5°C) considerably above freezing. This similarity in responses suggests that long‐term acclimation to a moderately cool temperature can produce similar results as a shorter‐term acclimation to a colder temperature. Furthermore, the presence of high light intensities under the HLC conditions may make a contribution given that long‐term acclimation to HLC conditions strongly induced *CBF1–3* expression in both ecotypes whereas LLC conditions did not.

The striking difference in the extent to which CBF1–3‐deficiency differentially impairs aspects of the acclimation process to HLC conditions in IT compared to SW is a key finding of the present study. While many genes previously defined as CBF1–3‐responsive genes did exhibit strongly reduced expression in both CBF1–3‐deficient lines, and may be associated with functions we did not characterize in this study, some genes instead exhibited trends matching those of photosynthetic acclimation and freezing tolerance of whole plants. For the latter genes, sw:*cbf123* compared to SW exhibited little or no difference as the result of CBF1–3‐deficiency, whereas it:*cbf123* exhibited strongly reduced expression compared to IT. The central features of the acclimation of plant form and function to HLC, that is, photosynthetic upregulation (and its associated morphological traits) as well as freezing tolerance, were only modestly impacted in sw:*cbf123* but were strongly impacted (especially in whole plants for the case of freezing tolerance) in it:*cbf123* compared to IT. These findings provide further indication for a role of CBF1–3‐independent pathways in HLC acclimation of photosynthesis and freezing tolerance and suggest a greater contribution of such pathways in SW.

Growth is yet another trait exhibiting differential regulation between SW and IT in the context of CBF1–3‐deficiency. Rosette size data indicate an obligatory role of CBF1–3 in growth depression under HLC conditions in IT but not in SW. While HLC‐grown rosettes of it:*cbf123* were larger relative to those of IT, those of sw:*cbf123* and SW were the same size. Ding et al. (2019) reported a regulatory link between CBF1–3 induction by chilling stress, posttranslational modification of EGR2, and whole‐plant changes in rosette growth. As noted, under HLC conditions *EGR2* was induced in both ecotypes (more strongly so in SW) and preferentially attenuated in it:*cbf123*. Given *EGR2*'s role in repressing leaf elongation (Bhaskara et al., 2017), this gene may contribute to the larger rosette size of it:*cbf123* relative to IT in HLC growth conditions. Future research should further clarify the role of CBF1–3 (in IT) and/other regulators (in SW) in inducing EGR2‐dependent growth depression under HLC.

Stunted growth in winter annuals has been interpreted as a protective response that minimizes freezing damage of the shoot under field conditions with variable temperatures (Eremina et al., 2016). CBF1–3 repression of rosette expansion in IT in HLC conditions with leaf temperatures of 16°C, and temperature of 8.5°C during the light period—well above the freezing temperatures that can damage foliar tissues—is consistent with a potential tradeoff between CBF‐mediated freezing tolerance and a cost of CBF induction to growth in cool environments with infrequent or only mild freezing events (Kang et al., 2013; Monroe et al., 2016). The IT ecotype contains a disrupted copy of *CBF2* and expression of SW *CBF2* in the IT ecotype background enhanced expression of the CBF target genes following a weeklong exposure to 4°C (Gehan et al., 2015). To test whether the loss of CBF2 activity in IT altered this growth versus survival tradeoff, in future work, the IT line expressing SW *CBF2* (Gehan et al., 2015) could be tested to see if it has a smaller rosette size relative to the IT background in HLC conditions.

In summary, the negligible to modest impact of CBF1–3 deficiency on photosynthetic upregulation and rosette morphology in the two ecotypes contrasts with the profound impact of CBF1–3 deficiency on freezing tolerance in both ecotypes. While photosynthetic upregulation was entirely independent of CBF1–3 in the SW ecotype, both photosynthetic upregulation and rosette morphology exhibited some dependency on CBF1–3 in the IT ecotype under HLC conditions.

#### 4.3.2 Role of paralog compensation

Could a closely related transcription factor be responsible (via paralog compensation) for the induction of CBF1–3‐target genes in CBF1–3‐deficient mutants in HLC? CBF1–3 belong to the ERF/AP2 A‐1 subfamily that includes three additional members located outside the *CBF1–3* gene locus in *A. thaliana* (Mizoi et al., 2012). These three other ERF/AP2 A‐1 subfamily members (*DDF1*, AT1G12610; *DDF2*, AT1G63030; *CBF4*, AT5G51990) were not expressed at detectable levels in leaf tissue of IT or SW under any of the four growth regimes in either the present study or a previous study (Park et al., 2018). This finding indicates that the latter transcription factors are either not involved or no longer active after long‐term acclimation.

#### 4.3.3 SW as a high‐light adapted ecotype

It was previously shown that SW responds with stronger upregulation of photosynthetic capacity and associated leaf features than IT to growth in high light under warm temperature (Stewart, Polutchko, Adams, & Demmig‐Adams, 2017). Based on the latter response, SW was classified as having a high‐light phenotype (Adams et al., 2016). Photosynthetic upregulation is a developmental process involving changes at the organelle, cell, tissue and whole plant levels (Hoshino et al., 2019; Yano & Terashima, 2004), and involves the integration of multiple regulatory pathways, including photoreceptors, photosynthesis‐related sugar and redox signals and phytohormone signals. For example, mutants in blue‐light photoreceptor signalling and foliar sucrose (Hoshino et al., 2019; Katagiri et al., 2016; Kozuka et al., 2011; López‐Juez et al., 2007) have an effect of similar magnitude in increasing leaf thickness in HL‐grown plants to those observed for CBF1–3‐dependent leaf thickening in the it:*cbf123* mutant under HLC. The sucrose synthase *SUS1* may contribute to the differential leaf thickening phenotype of SW and IT in HLC conditions via sucrose‐responsive leaf thickening (Katagiri et al., 2016) given that (a) it was induced in both ecotypes in HLC conditions, but more strongly in SW, and (b) its induction was unchanged in sw:*cbf123* but significantly attenuated in it:*cbf123* relative to each respective parental ecotype. Thus, its induction pattern in HLC closely mirrors the trends for leaf thickness reported here. In summary, the present findings suggest that light‐responsive signalling pathways with overlapping functions compensate fully for CBF1–3 deficiency in sw:*cbf123* with respect to upregulation of photosynthetic capacity and associated leaf features, which were unaffected in sw:*cbf123*, but significantly (albeit modestly) reduced in it:*cbf123*. The particularly pronounced photosynthetic upregulation in SW is presumably demanded by the continuously low temperatures at its high‐latitude site of origin, whereas the IT ecotype encounters intermittent cold spells (requiring oxidative‐stress mitigation) and can quickly resume photosynthetic activity upon return to milder temperatures (for temperature profiles at the respective sites of origin, see Adams et al., 2016).

#### 4.3.4 CBF1–3 and the nature of acclimation

The more pronounced photosynthetic upregulation in SW plants grown from seedling stage under HLC suggest an acclimation response directed at enhanced productivity in addition to mitigation of oxidative stress. Furthermore, the lesser excitation pressure (lower Q_A_ reduction state) in HLC‐grown plants of SW compared to IT represents a lesser trigger for further acclimatory adjustment and evidence of more complete acclimation to HLC conditions in SW compared to IT plants (see also Adams et al., 2013; Cphu et al., 2014; Cohu, Muller, Demmig‐Adams, et al., 2013, Cohu, Muller, Stewart, et al., 2013; Stewart, Polutchko, Adams, Cohu, et al., 2017). Additionally, as described above, genes involved in plant response to oxidative stress were consistently more strongly induced in IT relative to SW under HLC growth conditions. Rather than maximising excitation energy utilization for photosynthetic energy production and thereby minimising oxidant production, IT thus apparently employs multiple mechanisms that mitigate oxidative stress. CBF1–3 may play a prominent role in the mitigation of oxidative stress in IT, and presumably also during the initial stages of cold acclimation (Fowler & Thomashow, 2002) compared to completed acclimation in SW (see Park et al., 2018). This difference in transcriptional control in SW and IT may stem from evolution under the different environmental conditions at the sites of origin, where IT can presumably ‘wait out’ infrequent, short‐duration cold spells, while it is advantageous for SW to maintain productivity throughout long stretches of cool conditions. These contrasting strategies would be of interest for agriculture in locations with either short cold spells or continuously low temperatures.

In conclusion, several lines of evidence at the transcriptomic and physiological levels are consistent with the CBF1–3‐dependent pathway playing a disproportionately greater role under HLC in IT but not in SW. It should be noted that this trend was already evident in young plants and not only in more mature plants. The system of IT and SW, and their CBF1–3‐deficient mutants, can serve as a resource to further study CBF1–3‐regulated genes that mitigate oxidative stress before, or in the absence of, fully regained productivity as well as genes that remain active after productivity has been fully restored. In addition, CBF1–3‐independent pathways that contribute to full HLC acclimation can also be studied in the SW background. Tools for phenotyping and transcriptional profiling of Recombinant Inbred Line populations are available for these two populations (Ågren et al., 2013; Oakley et al., 2018).

## Supporting information


**Supporting information**.

**Supporting information**.

**Supporting information**.

## Data Availability

The data that support the findings of this study are available from the corresponding author upon reasonable request.
