# Supplementary material for: Genotype‐dependent contribution of CBF transcription factors to long‐term acclimation to high light and cool temperature
Source: Plant Cell Environ. 2021 Dec 6;45(2):392–411. doi: 10.1111/pce.14231 (PMC9299779; doi:10.1111/pce.14231)
Supplement: Supplementary file 1 — Supporting information. [file PCE-45-392-s001.pdf]

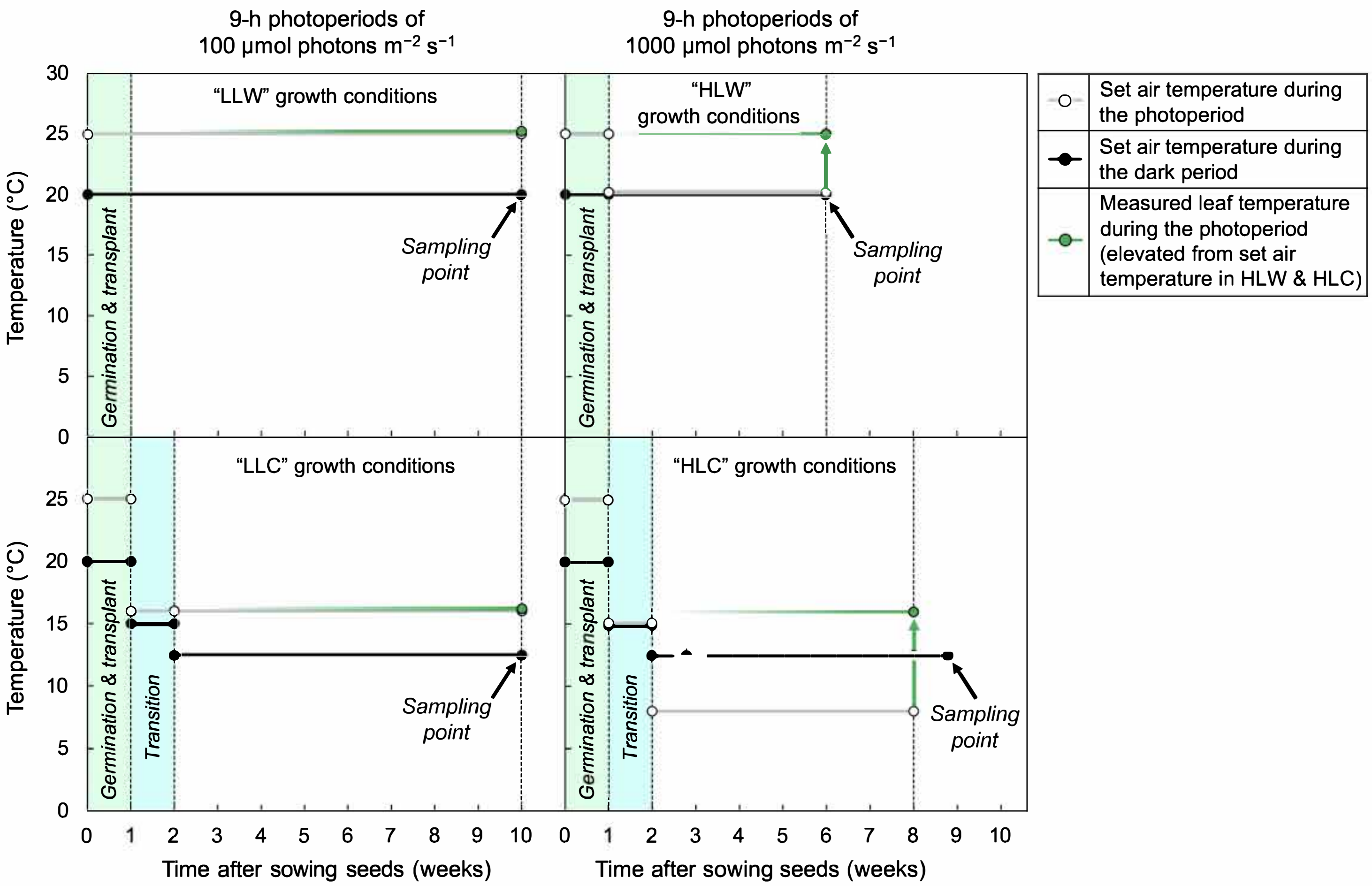
9-h photoperiods of 100 μmol photons m−2 s−1
9-h photoperiods of 1000 μmol photons m−2 s−1
"LLW" growth conditions
"HLW" growth conditions
"LLC" growth conditions
"HLC" growth conditions
Germination & transplant
Transition
Sampling point
Temperature (°C)
Time after sowing seeds (weeks)
Set air temperature during the photoperiod
Set air temperature during the dark period
Measured leaf temperature during the photoperiod (elevated from set air temperature in HLW & HLC)
