# Supplementary material for: Genotype‐dependent contribution of CBF transcription factors to long‐term acclimation to high light and cool temperature
Source: Plant Cell Environ. 2021 Dec 6;45(2):392–411. doi: 10.1111/pce.14231 (PMC9299779; doi:10.1111/pce.14231)
Supplement: Supplementary file 2 — Supporting information. [file PCE-45-392-s003.pdf]

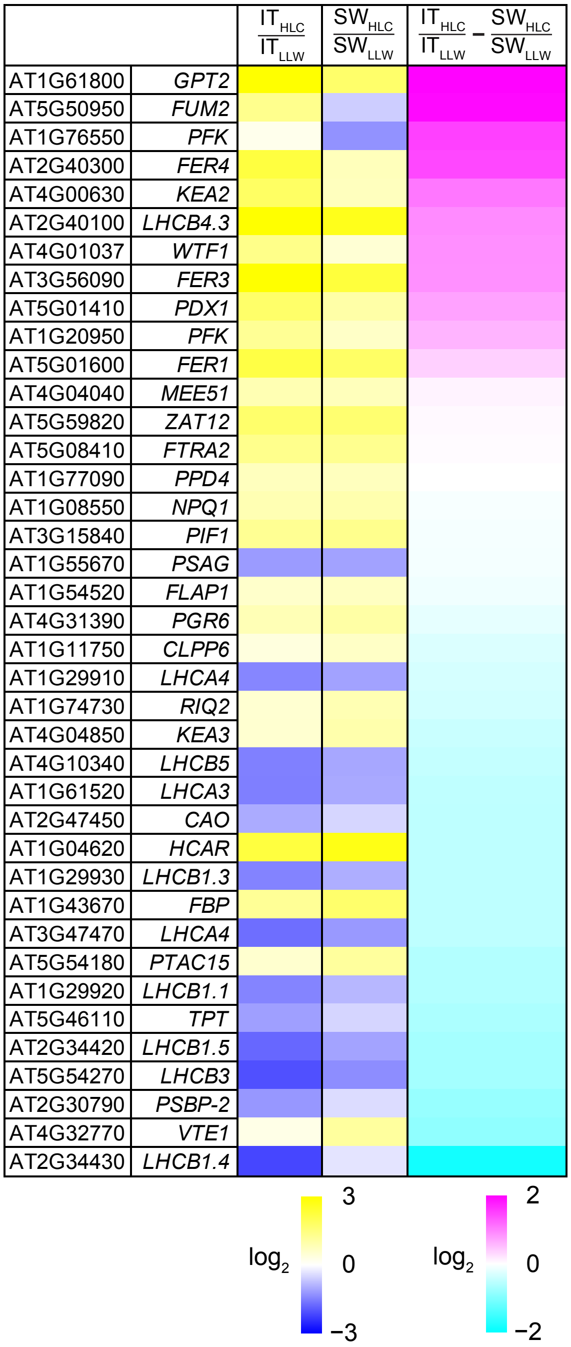

IT_HLC / IT_LLW
SW_HLC / SW_LLW
IT_HLC / IT_LLW – SW_HLC / SW_LLW
AT1G61800 GPT2
AT5G50950 FUM2
AT1G76550 PFK
AT2G40300 FER4
AT4G00630 KEA2
AT2G40100 LHCB4.3
AT4G01037 WTF1
AT3G56090 FER3
AT5G01410 PDX1
AT1G20950 PFK
AT5G01600 FER1
AT4G04040 MEE51
AT5G59820 ZAT12
AT5G08410 FTRA2
AT1G77090 PPD4
AT1G08550 NPQ1
AT3G15840 PIF1
AT1G55670 PSAG
AT1G54520 FLAP1
AT4G31390 PGR6
AT1G11750 CLPP6
AT1G29910 LHCA4
AT1G74730 RIQ2
AT4G04850 KEA3
AT4G10340 LHCB5
AT1G61520 LHCA3
AT2G47450 CAO
AT1G04620 HCAR
AT1G29930 LHCB1.3
AT1G43670 FBP
AT3G47470 LHCA4
AT5G54180 PTAC15
AT1G29920 LHCB1.1
AT5G46110 TPT
AT2G34420 LHCB1.5
AT5G54270 LHCB3
AT2G30790 PSBP-2
AT4G32770 VTE1
AT2G34430 LHCB1.4
log2
3
0
−3
log2
2
0
−2
